# Tracking the psychological and socio‐economic impact of the COVID‐19 pandemic in the UK: A methodological report from Wave 5 of the COVID‐19 Psychological Research Consortium (C19PRC) Study

**DOI:** 10.1002/mpr.1928

**Published:** 2022-06-27

**Authors:** Orla McBride, Sarah Butter, Jamie Murphy, Todd K. Hartman, Ryan McKay, Philip Hyland, Mark Shevlin, Kate M. Bennett, Thomas V. A. Stocks, Alex Lloyd, Jilly Gibson‐Miller, Liat Levita, Liam Mason, Anton P. Martinez, Frédérique Vallières, Thanos Karatzias, Richard P. Bentall

**Affiliations:** ^1^ Ulster University Coleraine UK; ^2^ University of Sheffield Sheffield UK; ^3^ University of Manchester Manchester UK; ^4^ Royal Holloway University of London Egham UK; ^5^ Maynooth University Maynooth Ireland; ^6^ University of Liverpool Liverpool UK; ^7^ University College London London UK; ^8^ Trinity College Dublin Dublin Ireland; ^9^ Napier University Edinburgh UK

**Keywords:** attrition, COVID‐19, longitudinal survey, mental health, psychological

## Abstract

**Objectives:**

The COVID‐19 Psychological Research Consortium (C19PRC) Study was established in March 2020 to monitor the psychological and socio‐economic impact of the pandemic in the UK and other countries. This paper describes the protocol for Wave 5 (March–April 2021).

**Methods:**

The survey assessed: COVID‐19 related experiences; experiences of common mental health disorders; psychological characteristics; and social and political attitudes. Adults who participated in any previous wave (*N* = 4949) were re‐invited to participate. Weights were calculated using a survey raking algorithm to ensure the longitudinal panel was nationally representative in terms of gender, age, and household income, amongst other factors.

**Results:**

Overall, 2520 adults participated. A total of 2377 adults who participated in the previous survey wave (November–December 2020) were re‐interviewed at Wave 5 (61.5% retention rate). Attrition between these two waves was predicted by younger age, lower household income, children living in the household, and treatment for mental health difficulties. Of the adults recruited into the C19PRC study at baseline, 57.4% (*N* = 1162) participated in Wave 5. The raking procedure re‐balanced the longitudinal panel to within 1.5% of population estimates for selected socio‐demographic characteristics.

**Conclusion:**

This paper outlines the growing strength of the publicly available C19PRC Study data for COVID‐19‐related interdisciplinary research.

## 1 INTRODUCTION

This report describes the design and conduct of the fifth wave of the COVID‐19 Psychological Research Consortium (C19PRC) Study, a longitudinal online survey of the UK adult population during the COVID‐19 pandemic. In this section, the context for the current survey wave is presented briefly, followed an outline of the key methodological issues associated with conducting this survey during the pandemic.

### 1.1 Context for C19PRC Study in March 2021

**FIGURE 1:**
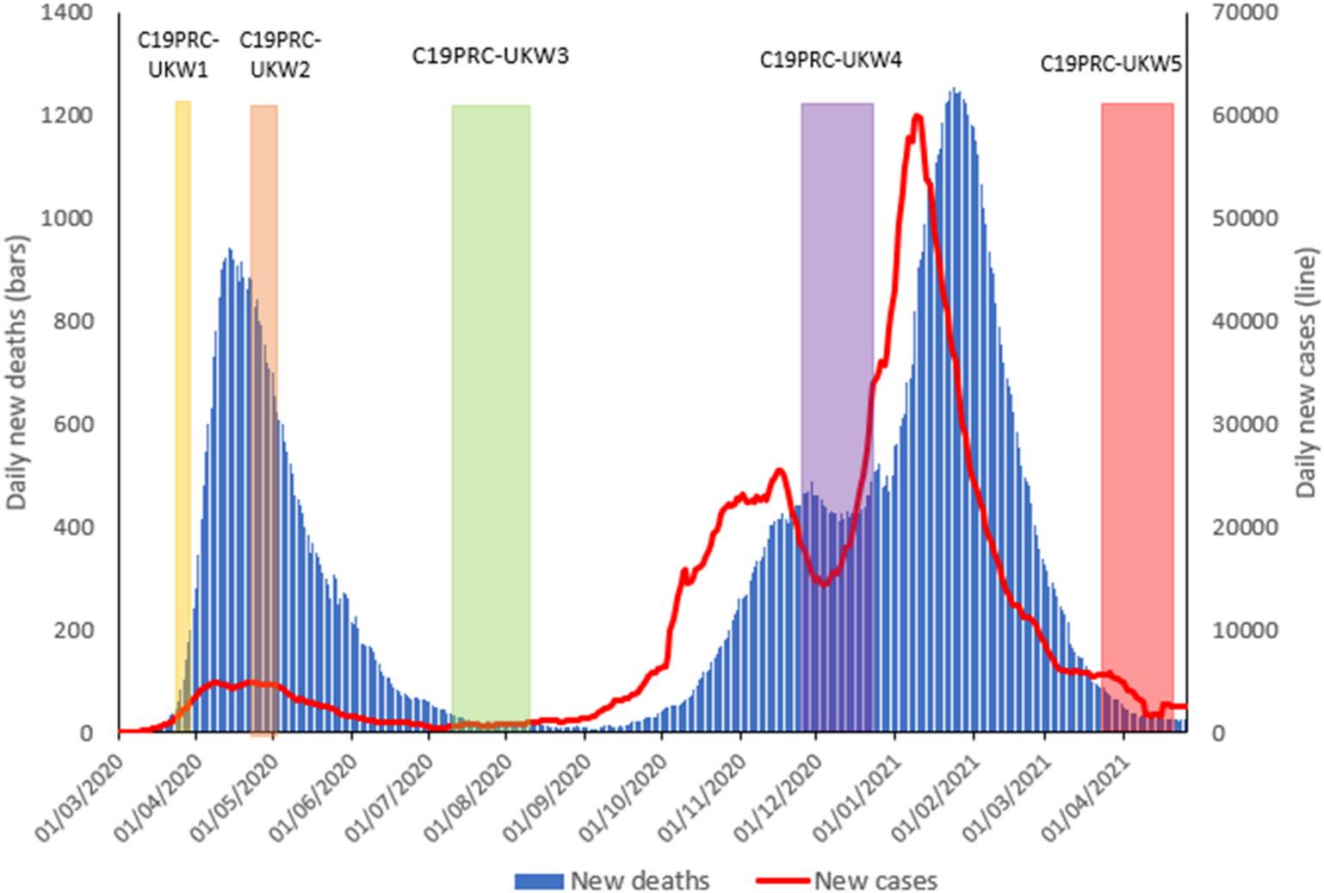
Graphical presentation of the number of daily COVID‐19 cases and deaths in the UK, sourced from Our World in Data, 2020, aligned to the COVID‐19 Psychological Consortium (C19PRC) Study survey waves. New daily deaths and cases depicted as 7‐day rolling average. C19PRC‐UKKW1 (baseline survey, March–April 2020); C19PRC‐UKW2 (second survey, April–May 2020); C19PRC‐UKW3 (third survey, July–August 2020), C19PRC‐UKW4 (fourth survey, November–December 2020), and C19PRC‐UKW5 (fifth survey, March–April 2021)

A ‘National Day of Reflection’ was held in the UK on 23 March 2021 to mark the one year anniversary of the nation's first COVID‐19 lockdown, and to pay respect to the ∼140,000 UK citizens who tragically died due to coronavirus in the previous 12 months (UK Government, 2021a). By this time, approximately 5000 new daily COVID‐19 cases were being reported in the UK (a substantial decrease from the ∼60,000 daily cases occurring at the peak of the second wave in January 2021—see Figure 1), and approximately 26 million COVID‐19 vaccination doses had been administered since the vaccination rollout commenced in the UK on 8 December 2020 (NHS England, 2021).

During March 2021, the UK Government enacted legislative changes to permit a gradual relaxation of the harsh lockdown restrictions which had been in place across the UK since late December 2020/early January 2021 (UK Government, 2021c). These included: (i) lifting the mandatory ‘stay at home’ rule, allowing the public to have socially‐distanced contact with individuals outside their household in outdoor spaces; (ii) children returned in‐person to school and childcare facilities; (iii) care home residents were able to receive regular in‐person visits from one person; and (iv) businesses offering outdoor facilities (e.g., tennis) were allowed to reopen (UK Government, 2021b). Directives to ‘work from home, where possible’ remained in place, however, and international travel was prohibited except for a limited number of permitted reasons (e.g., family death).

During 2020–21, the UK experienced notable social and economic disruption, attributable not only to the pandemic, but also to recent upheavals relating to the end of the Brexit transition period on 31 December 2020 (De Lyon & Dhingra, 2021). For example, the national lockdowns are estimated to have cost the UK economy £251bn in the value of goods and services (Miley, 2021), while the gross domestic product in January 2021 was 9% lower than the pre‐pandemic level a year earlier (Office for National Statistics, 2021b). Approximately 6.1 million people, or 19% of UK businesses' workforce, remained on furlough leave during February and March 2021 (Office for National Statistics, 2021a). Moreover, concern about the impact of recurrent lockdowns on the nation's mental health and wellbeing, a consistent issue at the forefront of public and academic debate since the earliest stages of the pandemic, persisted (Mental Health Foundation, 2021).

It is against this backdrop that the fifth UK survey wave of the C19PRC Study (hereafter C19PRC‐UKW5) commenced on 22 March 2021 to coincide with the anniversary of the UK's first national lockdown. The C19PRC Study has followed a large, nationally representative sample of UK adults over multiple survey waves from the beginning of the first national lockdown in the UK, through the summer and winter of 2020, and into spring 2021 (see Figure 1). The main objective of the C19PRC Study has been to investigate trends in mental health outcomes (i.e., experiences of major depression, anxiety, and pandemic related traumatic stress) for the UK adult general population over time, and to determine how various psychological, socio‐economic, and political factors have influenced these trends, whilst accounting for the wider national context in which the pandemic has been unfolding. Detailed methodological reports for these survey waves are available elsewhere (McBride, Butter, Murphy, Shevlin, Hartman, Bennett, et al., 2021; McBride, Butter, Murphy, Shevlin, Hartman, Hyland, et al., 2021; McBride, Murphy, et al., 2021). Parallel studies have also been conducted in the Republic of Ireland, Spain, and Italy (Bruno et al., 2021; Spikol et al., 2021; Valiente et al., 2021).

### 1.2 Methodological issues relating to online survey research during pandemic

The C19PRC Study survey waves are being designed and conducted during an unprecedented time for survey fieldwork. As we have previously discussed (McBride, Murphy, et al., 2021), the four main methodological challenges or issues relate to: (i) the use of quota‐based sampling to recruit participants from existing opt‐in market research survey panels, as opposed to probability‐based sampling; (ii) a lack of pre‐pandemic data on C19PRC Study participants to assess change in core study outcomes (e.g., common mental health conditions) pre‐to‐post lockdown; (iii) mode of administration; and (iv) managing attrition. We will briefly summarise each of these challenges next.

With respect to surveying the UK adult population during the pandemic, two general approaches were available to researchers in March 2020. The first is that specific research teams already in the field were able to *repurpose existing surveys* to capture important COVID‐19 related data from their study's participants. For example, many of the established UK‐based cohort studies (e.g., Understanding Society; Millennium Cohort, etc.) fielded COVID‐19 waves to collect new data from existing cohort members who had been recruited using probability‐based sampling techniques before the pandemic (Patel et al., 2022). The superiority of probability‐based samples over non‐probability quota based samples is well‐acknowledged (Pierce, McManus, et al., 2020). An additional strength of this approach is the availability of ‘pre‐pandemic’ data, often spanning many years or even decades, to detect meaningful change more readily in respondents' lives as a result of their experiences during the pandemic (Pierce, Hope, et al., 2020). Moreover, research teams working with existing cohorts were also ideally positioned to offer different modes of survey administration (e.g., telephone or web‐based interviews), and to assess the likely impact of these different modes on the quality and completeness of data collection (Burton et al., 2020). Attrition in these repurposed surveys was generally managed using post‐survey weighting procedures (Benzeval et al., 2021).

In the second approach, research teams set about designing new studies to collect COVID‐19 specific data from general population samples. As we previously discussed (McBride, Butter, Murphy, Shevlin, Hartman, Hyland, et al., 2021), many of these studies: (i) were established hastily and limited to one or two waves of online data collection; (ii) relied on short screener‐type questionnaires to measure general wellbeing or psychological distress, as opposed to gold standard instruments for assessing common mental disorders; and (iii) comprised of relatively small sample sizes recruited via opportunistic sampling methods, the composition of which did not represent the socio‐demographic characteristics of the UK general adult population.

The C19PRC Study is one of the longest running newly‐established COVID‐19 surveys in the UK. Funded by the UKRI Economic and Social Research Council (ESRC), the C19PRC Study was designed to address many of the limitations of survey fieldwork encountered during the pandemic. An important feature of our Consortium's work is the production of detailed methodological papers for each survey wave in which we document the survey design and content, as well as the challenges and outcomes associated with conducting the survey wave at a specific point in the pandemic. To our knowledge, this is not common practice for other longitudinal surveys conducted during the pandemic; indeed, the absence of detailed methodological reports for other dedicated COVID‐19 studies makes it challenging to compare surveys conducted during the pandemic in terms of fieldwork outcomes (e.g., retention rates). We argue this is a key strength of the C19PRC Study data, which is available for secondary use of the data via the Open Science Framework.

Here, we offer a brief summary of the characteristics of the C19PRC Study design for interested users of the data. Although the C19PRC Study recruited using quota‐based non‐probability sampling methods, the baseline sample was large and representative of the UK population on a wide range of socio‐demographic characteristics. The collection of robust mental health data using detailed measures of common mental health conditions was prioritised at each survey wave. Concerted efforts were made to re‐contact and re‐engage all study participants at each wave post‐baseline to encourage them to participate. Tailored communications were sent to participants to remind them about their previous engagement with the study and reassuring them of the importance of their valuable contribution to the main goal of the study (i.e., tracking the general public's experience of the pandemic over time). Approximately six‐in‐ten of baseline respondents returned at each follow‐up wave. Levels of attrition were low, with only 15% of baseline respondents completely lost to follow‐up by the fourth wave (McBride, Butter, Murphy, Shevlin, Hartman, Bennett, et al., 2021). Two specific approaches were taken to address attrition: (i) sample replenishment procedures were conducted regularly to ‘top‐up’ gaps in quotas (with respect to age, gender, and household income); and (ii) post‐survey weighting was conducted to ensure the longitudinal panel followed from baseline was representative of the UK general adult population. Finally, booster sampling was conducted by UK country to ensure that there was sufficiently large sample sizes to conduct robust between‐country analyses.

The availability of the fifth wave of the C19PRC Study offers an ideal opportunity to further study attrition processes for a large, internet‐based panel of adults recruited and followed‐up during a turbulent historical event. This paper has three main aims: (i) to describe the prevalence of common mental disorders among participants in the C19PRC‐UKW5 sample, as well as the sample's socio‐demographic characteristics and specific experiences relating to the pandemic which were pertinent issues at the time this wave was conducted (e.g., self‐isolation, diagnosis of COVID‐19, and vaccination status); (ii) to examine patterns of attrition in the C19PRC Study by this fifth wave, and test whether these could be predicted by respondents' mental‐health attributes, psychological characteristics, and socio‐demographic factors; and (iii) to conduct and assess weighting procedures to manage attrition in the longitudinal panel.

## 2 METHOD

A Checklist for Reporting Results of Internet E‐Surveys (CHERRIES) (Eysenbach, 2004) for this survey wave is available in the Supplementary Tables Document (see Table S1).

### 2.1 C19PRC‐UKW5: Fieldwork procedures

#### 2.1.1 Fieldwork organisation overview and strategy

The survey company Qualtrics conducted the fieldwork for C19PRC‐UKW5. Qualtrics partners with over 20 online sample providers to supply a network of diverse, quality respondents to their worldwide client base. To date, the company has completed ∼15,000 projects across ∼2500 universities worldwide.

As described elsewhere (McBride, Butter, Murphy, Shevlin, Hartman, Bennett, et al., 2021), at the previous survey wave, C19PRC‐UKW4, which was conducted during November–December 2020, a complex booster‐sampling strategy was employed to (i) recruit new respondents into the panel by oversampling in each of the devolved UK nations (Scotland, Wales, and Northern Ireland) so that sizeable country‐specific sub‐samples would be available to facilitate robust, between‐country comparisons; and (ii) replenish the sample with new recruits according to baseline quotas (i.e., age, gender, and household income) to deal with attrition across previous waves to ensure the main longitudinal panel remains representative of the UK adult population (with respect to these characteristics). This strategy increased the panel sample from 2025 to 4949 adults. Funding budgetary constraints at this stage in the C19PRC study meant that a maximum of 3600 respondents could be re‐interviewed at C19PRC‐UKW5, and so a decision was made to re‐contact all adults who participated in the previous wave (C19PRC‐UKW4) first, as a priority. Two recruitment Phases were designed to achieve this aim (described next).

#### 2.1.2 Procedure

Online fieldwork for C19PRC‐UKW5 commenced on 22 March 2021, approximately three months after the completion of C19PRC‐UKW4 and one‐year post the baseline survey.

In Phase 1 (24 March–20 April 2021), Qualtrics re‐contacted all adults who participated in the previous survey wave (C19PRC‐UKW4) (*N* = 3867) via email, SMS, or in‐app notifications and invited them to participate further in this survey, with invitations tailored to remind adults of their participation in a previous survey wave(s).

In Phase 2 (8–20 April 2021), participants who had completed any previous wave except C19PRC‐UKW4 (*N* = 1082) were recontacted and invited to participate in the fifth wave.

#### 2.1.3 Informed consent process

As in previous waves, participants were informed, that their data would be treated in confidence, that geolocating would be used to determine the area in which they lived (in conjunction with their residential postcode stem), and of their right to terminate participation at any time. Participants were also informed that some topics might be sensitive or distressing (e.g., self‐harm/suicide content). Information about how their data would be stored and analysed by the research team was also provided. Participants were also informed that they would be re‐contacted at a later date to invite them to participate in subsequent survey waves. Participants provided informed electronic consent prior to completing the survey and were directed to contact the NHS website upon completion if they had any concerns about COVID‐19, and emotional support services if they had been negatively impacted by any of the questions asked during the survey.

#### 2.1.4 Compliance with General Data Protection Regulation (GDPR)

Participants are informed that C19PRC data will be stored confidentially in line with GDPR. When the study data is deposited with the UK Data Service and the Open Science Framework (OSF), location data is removed and replaced with relevant socioeconomic summary data (e.g., area‐level deprivation and population density data). All other personal data is also removed.

#### 2.1.5 Quality control

Qualtrics deliver high‐quality survey data from online survey panels and conduct multiple validation checks on the C19PRC survey data. First, the survey is piloted (‘soft launch’; *n* = 50) prior to the fieldwork going live (‘full launch’) to rectify sequencing/coding errors and omissions prior to the full launch. The soft launch also calculates the median survey completion time, providing an opportunity to tailor the content to ensure the median survey time does not exceed 30 min; this is important to minimise respondent burden and maximise participation over time. A soft launch for C19PRC‐UKW5 was conducted using a new general population sample of (*n* = 51) on 22 March 2021, and the median survey completion time was 19 min 34 s. Participants in the soft launch are excluded from the final sample for the survey wave.

### 2.2 Measures

Table 1 provides an overview of the C19PRC‐UKW5 survey content (see Supporting Information S1 for details of all measures administered).

**TABLE 1:** Overview of content^a^ of C19PRC Study Wave 5 (Phases 1 & 2), United Kingdom (UK), (March–April 2021)

Theme	Content	C19PRC Wave 5
		Phases 1 & 2
Demographics	Age, gender, country of residence, type of secondary education, religion, sexual orientation, current relationship status, previous relationships, partner's ethnicity, economic activity, key/essential worker status, perceived social rank	X
Housing characteristics	Living alone^b^	X
	Number of adults living in household^b^	X
	Parental and children in the home status	X
	Housing tenure^b^	X
	Residential details (type of property; number of bedrooms; urbanicity)^b^	X
	Outdoor/garden space	X
	Indoor residence characteristics (space, privacy, broadband)	X
	Belongingness in neighbourhood	X
Household finances	Estimated annual gross household income	X
	Change in monthly household income during pandemic	X
	Use of savings/increasing debt during pandemic	X
	Made saving due to pandemic	X
	Purchases from pandemic savings	X
	Concern over household finances being negatively affected due to pandemic	X
	Perceived future financial security	X
	Receiving benefits	X
	Perceived future job loss/income security	X
	Difficulty paying bills	X
	Food insecurity: Past year	X
Working hours	Number of hours worked weekly pre/post pandemic	X
	Number of hours would like to be working	X
Health conditions and behaviours	Currently pregnant—self (partner)	X
	Number of weeks pregnant, if applicable	X
	Currently pregnant—immediate family member	X
	Family planning	X
	Self‐rated health	X
	Health service use	X
	Weight (classification and weight change)	X
	Alcohol use	X
	Sleep problems: *Sleep Disorders Symptom Checklist‐17 (SDS‐CL‐17)* (Klingman et al., 2017)	X
COVID‐19	Anxiety‐level relating to COVID‐19	X
	Confidence in response to COVID‐19 pandemic	X
	Perceived threat of COVID‐19	X
	Perceived individual risk contracting COVID‐19 over next month	X
	Perceived severity of COVID‐19 symptoms if infected/reinfected	X
	Experiences of self‐isolation	X
	Experiences of children in the home self‐isolating	X
	Experience of being infected with COVID‐19 (self and family member/friend)	X
	Knowing someone close (family member/friend) who has tested positive for COVID‐19	X
	Bereavement due to COVID‐19	X
	Behaviour—engagement with social distancing/social contact	X
	Behaviour—engagement with hygiene practices	X
	Capability, opportunity and motivation to engage with social distancing	X
	Capability, opportunity, and motivation to take a COVID‐19 vaccine^c^	X
	COVID‐19 vaccine acceptability (self) (if vaccinated, if intending to vaccinate, preference for vaccine)	X
	COVID‐19 vaccine acceptability (child)	X
	Family and friend COVID‐19 vaccination and reaction	X
	Beliefs about vaccines (safety and effectiveness)	X
	COVID‐19 vaccine conspiracy beliefs	X
	Science conspiracy beliefs	X
	Support/opposition for mandatory vaccination	X
	Predicted course of the pandemic	X
	Perceived risk of future pandemic	X
	Life after the pandemic (increase/decrease in behaviours)	X
Mental health	Depression: *Patient Health Questionnaire‐9* (Kroenke et al., 2001)	X
	Anxiety: *Generalised Anxiety Disorder Scale‐7* (Spitzer et al., 2006)	X
	Traumatic stress *International Trauma Questionnaire* (Cloitre et al., 2018)	X
	Prolonged grief disorder: *International Prolonged Grief Disorder Scale* (Killikelly et al., 2020)	X
	Self‐harm, suicidal thoughts, and suicide attempts	X
	Defeat and entrapment: *The Short Defeat and Entrapment Scale* (Griffiths et al., 2015)	X
	Perceived burdensomeness & thwarted belongingness: *Interpersonal Needs Questionnaire* (Van Orden et al., 2012)	X
	Mania: *Mood Disorders Questionnaire* (Hirschfeld et al., 2000)	X
	Psychotic experiences: *Psychosis Screening Questionnaire* (Bebbington & Nayani, 1995)	X
	Treatment for mental health difficulties	X
Psychological factors	Loneliness: *Loneliness Scale* (Hughes et al., 2004)	X
	Hopefulness: *Brief‐H‐Positive* (Fraser et al., 2014)	X
	Happiness: Degree of happiness yesterday	X
	Daily functioning and wellbeing at home—Helpful and harmful activities	X
	Wellbeing: *Warwick‐Edinburgh Mental Wellbeing Scale (WEMWBS, short 7‐item version)* (Stewart‐Brown et al., 2009)	X
	Social engagement/contact	X
	Adverse childhood experiences: *ACE Scale* (Felitti et al., 1998)	X
Parenting^d^	Parenting style: *Parenting Scale Short Form (PS‐8)* (Kliem et al., 2019)	X
	Home‐schooling	X
	Parental warmth and criticism	X
Socio‐political views/related behaviours	Authoritarianism: *Very Short Authoritarianism Scale* (Bizumic & Duckitt, 2018) and *Left‐Wing Authoritarianism Index* (Costello et al., 2020)	X
	Hindsight attitudes towards Brexit	X
	Perceived impact of Brexit on UK	X
	Family/friends disharmony: Political and COVID‐19 beliefs	X
	Future voting behaviour—General Election	X
Trust	Institutions	X

^a^ Refer to Supporting Information S1 for detailed information on all study measures.

^b^ These items only asked if respondent reported that their living situation had changed since last completing the survey.

^c^ Asked only to those who reported that they did not intend or were unsure if they would accept a COVID‐19 vaccine.

^d^ Questions in this module were only presented to parents of children under 18/adults with children under 18 in the home.

### 2.3 Ethical approval

Ethical approval for the project was provided by the University of Sheffield (Reference number 033759).

### 2.4 Data analysis plan and weighting procedures

Five sets of analyses are presented.

First, re‐contact rates at C19PRC‐UKW5 were calculated for Phase 1 and Phase 2, and patterns of respondent participation in previous waves by phase were described and compared.

Second, the socio‐demographic, mental health, and COVID‐19 related characteristics of the cross‐sectional sample surveyed at C19PRC‐UKW5 are presented.

Third, a binary logistic regression model was estimated to assess the extent to which participation at C19PRC‐UKW5 could be predicted by a range of socio‐demographic factors, mental health conditions, and psychological factors assessed at the previous wave.

Fourth, the process and outcome of post‐stratification survey weighting for this longitudinal panel is detailed. As per previous waves, survey raking or sample‐balancing was conducted using the ‘anesrake’ package in R (Pasek & Pasek, 2018). Raking is one common method of adjusting survey data to ensure that the distribution of the characteristics of a given sample closely mirror the known population distribution. In practice, this means the baseline sampling quotas for age, gender, and household income, as well as the baseline proportions achieved for ethnicity, urbanicity, household composition, and being born or raised in the UK, were imposed on the sample of respondents returning from baseline at C19PRC‐UKW5, and the raking algorithm was conducted to produce, and iteratively adjust, a weight value for each case in the sample until the sample distribution aligned with the population distribution for the chosen characteristics (DeBell & Krosnick, 2009).

And fifth, the characteristics of the core longitudinal panel (i.e., those involved in the C19PRC Study since baseline) participating in C19PRC‐UKW5 are described.

#### 2.4.1 Study variables

Given the broad focus of the C19PRC Study in understanding the impact of the pandemic on the UK adult general population, a wide range of socio‐demographic, economic, and psychological factors were selected to describe the characteristics of the sample participating at this wave, as well as to identify predictors of attrition from the previous wave (C19PRC‐UKW4): gender (females vs. males); age (18–24 years olds vs. 25–34 years, 35–44 years, 45–54 years, 55–64 years, and 65+ years groups); household income (≤£15,490 per annum vs. £15,491–£25,340, £25,341–£38,740, £38,741–£57,903, and ≥£57,931 bands); economic activity (employed vs. other); ethnicity (White vs. other); born in UK (yes vs. no); household composition (living alone vs. other; children <18 years living in household vs. other); probable depression diagnosis (score of ≥10 on the *Patient Health Questionnaire‐9* vs. other); probable generalised anxiety diagnosis (score of ≥10 on the *Generalised Anxiety Disorder‐7* vs. other); probable PTSD diagnosis (using the *International Trauma Questionnaire's* diagnostic algorithm for PTSD caseness relating to experience of COVID‐19 vs. other); mental health treatment (current or past treatment for mental health problems vs. other); loneliness (score of ≥6 on the *Loneliness Scal*e); neuroticism (total score on the neuroticism subscale of the *Big‐Five Inventory‐10*); paranoia (total score on the *Persecution and Deservedness Scale*); Conspiracy mentality (total score on the *Conspiracy Mentality Questionnaire*); hopefulness (total score on the *Brief‐H‐Pos Scale*) and COVID‐19 anxiety (total score on single item indicator).

In addition, these variables (same categorisation as above, unless otherwise specified) were used to describe the attrition analyses for longitudinal panel (recruited at baseline) participating in C19PRC‐UKW5: gender; age; household income; urbanicity (city vs. suburb/town/rural); ethnicity; birthplace (born or raised in UK vs. other); household composition; depression; anxiety; and PTSD.

Finally, additional variables measured at C19PRC‐UKW5 were used to describe the C19PRC‐UKW5 cross‐sectional sample: relationship status (married, civil partnership, cohabiting, committed relationship, single); sexual orientation (heterosexual, gay/lesbian/homosexual, bisexual, other); country of residence (England, Wales, Scotland, Northern Ireland); experience of self‐isolation during pandemic (yes/no), experience of COVID‐19 (yes, no, not sure), received a COVID‐19 vaccine (yes/no—a distinction between first and second doses was not assessed at this wave given the early stage of the vaccination rollout in the UK in March 2021), and perspective on worst of pandemic (behind us, happening now, ahead of us).

## 3 RESULTS

**FIGURE 2:**
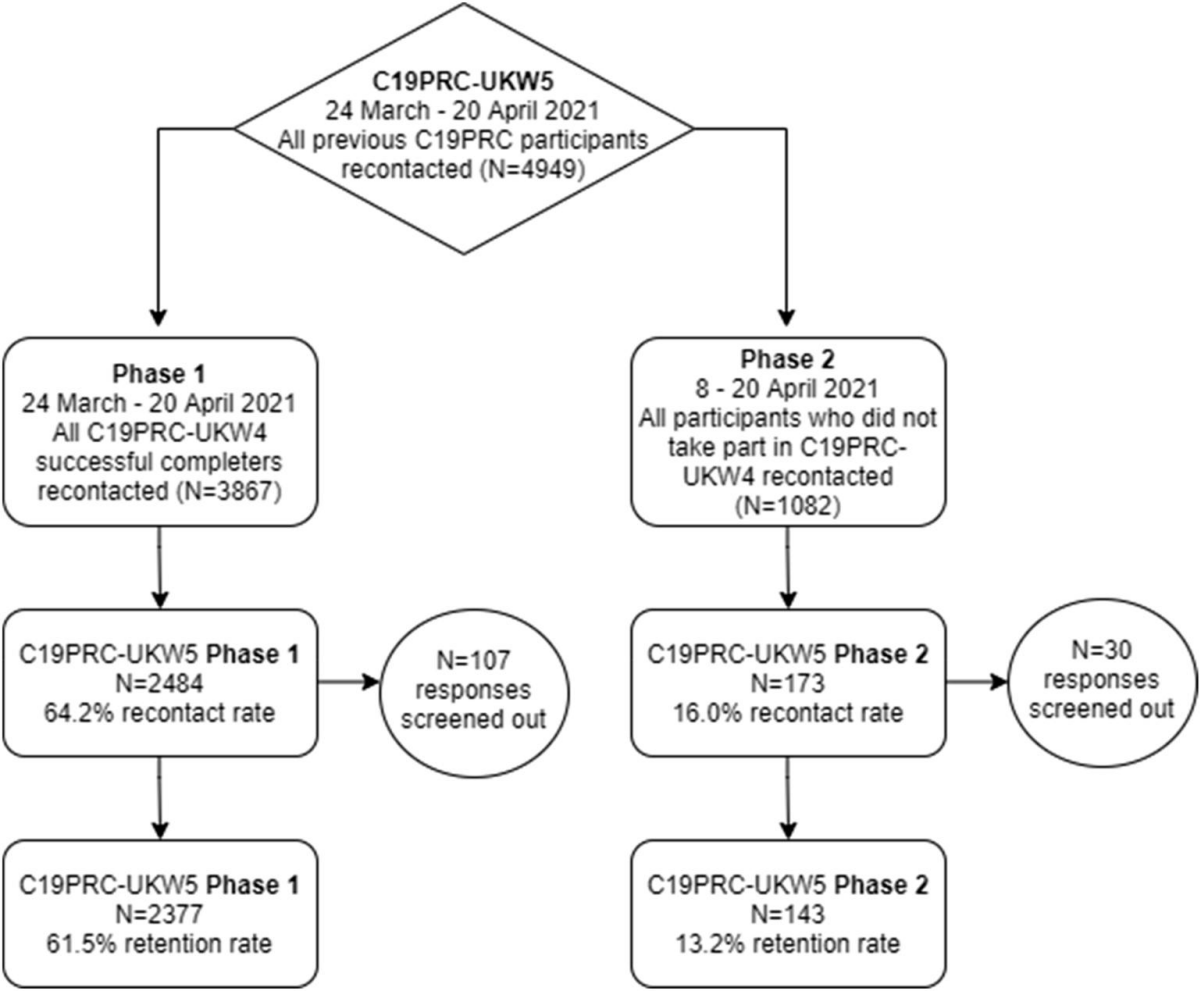
Flow chart for participation in the fifth wave of the COVID‐19 Psychological Research Consortium (C19PRC) Study, March–April 2021. Responses were screened out due to a failure to meet quality control checks

Figure 2 illustrates the outcome of recruitment of C19PRC‐UKW5, Phase 1 and Phase 2. The median survey completion time for both Phases was 31 min 28 s.

### 3.1 Outcome of recruitment at Phase 1 and Phase 2, by participation in panel to date

At Phase 1, 3867 adults were eligible to participate in C19PRC‐UKW5 having completed the survey at the previous wave, and 2377 were successfully reinterviewed (61.5% recontact rate). Table 2 (Panel A) illustrates C19PRC survey wave participation for Phase 1 non‐responders compared to responders. The majority of Phase 1 non‐responders (77.2%) entered the survey at the previous wave and were not re‐interviewed at this point of first follow‐up; the remainder (22.8%) had participated in two or more C19PRC survey waves, but only 6.3% had participated in all previous waves. In contrast, 28.5% of Phase 1 responders completed all previous survey waves, with the remainder (71.5%) completing two or more survey waves.

**TABLE 2:** Patterns of participation (green) and non‐participation (red) by respondents in C19PRC survey waves by the fifth wave (C19PRC‐UKW5, March–April 2021) Panel A (C19PRC‐UKW5; Phase 1)

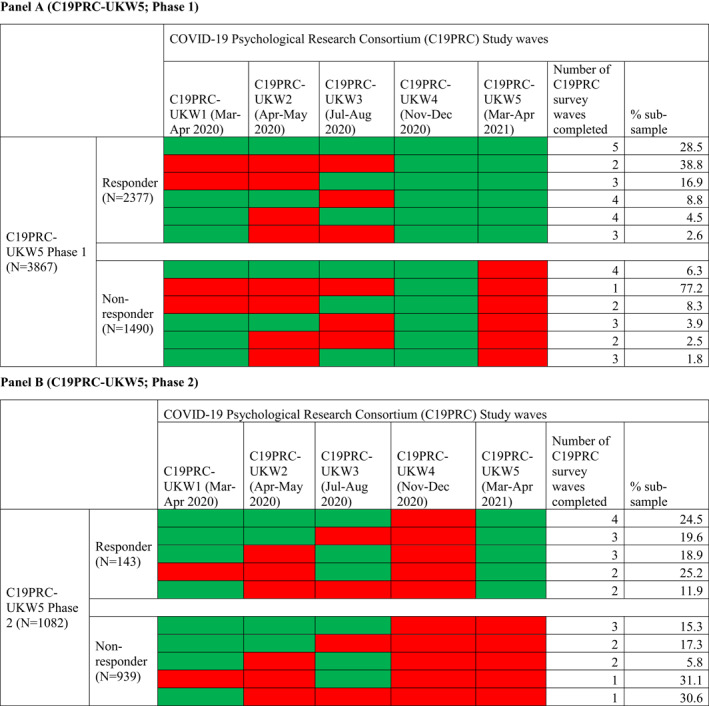

Table 2 (Panel B) illustrates C19PRC survey wave participation for Phase 2 non‐responders compared to responders. The majority of Phase 2 non‐responders (*N* = 580; 61.7%) participated in only one previous wave, either at baseline (C19PRC‐UKW1, March–April 2020) or at the third wave (C19PRC‐UKW3, July–August 2020), which was the first wave in which sample replenishment was conducted (McBride, Butter, Murphy, Shevlin, Hartman, Hyland, et al., 2021); the remainder (38.3%) had participated in two or three previous waves. Almost one‐quarter (24.5%) of Phase 2 responders participated in all C19PRC study waves except C19PRC‐UKW4, with the remainder participating in any two or three of the waves at this point in the study.

### 3.2 Characteristics of cross‐sectional sample: C19PRC‐UKW5

Table 3 presents a description the C19PRC‐UKW5 cross‐sectional sample (*N* = 2520) with respect to socio‐demographic characteristics and common mental disorders. Overall, despite a preponderance of older adults (66.2% of the sample were aged 45 years or older), there was good representation across gender, household income, economic activity, relationship status, sexual orientation, household composition, urbanicity, and country of residence. For the core C19PRC mental health study outcomes (i.e., probable diagnoses of major depression, generalised anxiety disorder, and COVID‐19 PTSD), the proportions of adults meeting caseness for these conditions when surveyed 1 year into the pandemic were 21.6%, 16.9%, and 11.9%, respectively.

**TABLE 3:** Socio‐demographic characteristics and prevalence of mental health disorders of the C19PRC‐UKW5 cross‐sectional sample (*N* = 2520) (March–April 2021)

Respondent characteristics (C19PRC‐UKW5)			*N* (%)
Socio‐demographic	Gender	Male	1267 (50.3%)
		Female	1246 (49.4%)
		Other	7 (0.3%)
	Age group (years)	18–24 years	97 (3.8%)
		25–34 years	335 (13.3%)
		35–44 years	419 (16.6%)
		45–54 years	516 (20.5%)
		55–64 years	593 (23.5%)
		65+ years	560 (22.2%)
	2019 household income	≤£15.490	481 (19.1%)
		£15,491–£25,340	465 (18.5%)
		£25,341–£38,740	563 (22.3%)
		£38,741–£57,903	518 (20.6%)
		≥£57,931	493 (19.6%)
	Economic activity	Employed (incl. full or part‐time, self‐employed, and furloughed)	1475 (58.5%)
		Other	1045 (41.5%)
	Relationship status	Married	1262 (50.1%)
		Civil partnership	9 (0.4%)
		Cohabiting	295 (11.7%)
		Committed relationship	177 (7.0%)
		Single	777 (30.8%)
	Sexuality	Heterosexual	2288 (90.8%)
		Gay/lesbian/homosexual	115 (4.6%)
		Bisexual	75 (3.0%)
		Other/prefer not to say	42 (1.6%)
	Household characteristics	Single adult household (i.e., living alone)	614 (24.4%)
		Other	1906 (75.6%)
		Children under 18 years living in household	517 (20.5%)
		Other	2003 (79.5%)
	Urbanicity	Suburb/Town/Rural	2040 (81.0%)
		City	480 (19.0%)
	Country of residence	England	1433 (56.9%)
		Wales	432 (17.1%)
		Scotland	393 (15.6%)
		Northern Ireland	262 (10.4%)
Mental health conditions and treatment	Depression (PHQ‐9)	Caseness met	545 (21.6%)
		Not met	1975 (78.4%)
	Anxiety (GAD‐7)	Caseness met	425 (16.9%)
		Not met	2095 (83.1%)
	COVID‐19 PTSD	Caseness met	299 (11.9%)
		Not met	2221 (88.1%)
	Treatment history	Never received	1791 (71.1%)
		Received in the past	439 (17.4%)
		Currently receiving	172 (6.9%)
		Other	118 (4.6%)
COVID‐19 related experiences and perspectives	Self‐isolated during pandemic	Yes	613 (24.3%)
		No	1907 (75.5%)
	Had COVID‐19	Yes	190 (7.5%)
		No	2164 (85.9%)
		Not sure	166 (6.6%)
	Vaccinated	Yes	1627 (64.6%)
		No	893 (35.4%)
	Worst of pandemic	Behind us	1601 (63.5%)
		Happening now	648 (25.7%)
		Ahead of us	271 (10.8%)

*Note*: No weighting variable generated for C19PRC‐UKW5 cross‐sectional sample.

Given that the baseline sampling quotas and subsequent sample replenishment to ‘top‐up’ the sample to quotas during the C19PRC Study were not interlocking, we further examined the characteristics of the C19PRC‐UKW5 cross‐sectional sample by gender and age group (see Table S2). The gender distribution across the age bands varied, with higher proportions of females in the younger age bands (18–44 years), and higher proportions of males in the older age bands (45–65+ years). Particularly noteworthy is that within the 18–24 years and 25–34 years age bands, 61.9% and 73.1% of these participants were female (respectively), whereas only 44.5% of the 55–64 years and 38.9% of the 65+ year age groups were female.

### 3.3 Attrition analysis at C19PRC‐UKW5

Table 4 presents the results of the binary logistic regression analysis predicting participation in C19PRC‐UKW5, based on socio‐demographic, mental health, and psychological characteristics data collected at the previous wave. Adults in every age group compared to those aged 18–24 were at higher odds of participating in the fifth wave (ORs ranged from 2.58 to 5.43). Adults in the highest household income bracket were at increased odds of participating (OR = 1.68; 95%CI 1.28–2.20) compared to those in the lowest household income bracket. Adults born in the UK had higher odds for participating in this fifth wave (OR = 1.33; 95%CI 1.01–1.74) compared to those born outside the UK. Respondents with children living in the household were at lower odds of participating compared to those without children (OR = 0.80; 95%CI 0.67–0.95).

**TABLE 4:** Respondent characteristics at Phase 1 C19PRC‐UKW4, November–December 2020 predicting participation at C19PRC‐UKW5 March–April 2021 (*N* = 3867)

		Responder at C19PRC‐UKW5 having participated in previous wave (*N* = 2377) versus non‐responders (*N* = 1490)
C19PRC‐UKW4 characteristics		Odds ratio (95% confidence interval)
Gender^a^	Male	1
	Female	1.12 (0.97–1.30)
Age group (years)	18–24	1
	25–34	2.58 (1.92–3.47)***
	35–44	3.52 (2.59–4.78)***
	45–54	4.75 (3.50–6.45)***
	55–64	5.42 (4.00–7.38)***
	65+	5.43 (3.89–7.59)***
2019 household income	≤£15,490	1
	£15,491–£25,340	1.10 (0.89–1.37)
	£25,341–£38,740	1.16 (0.93–1.44)
	£38,741–£57,903	1.19 (0.94–1.50)
	≥£57,931	1.68 (1.28–2.20)***
Employment	Employed	1.02 (0.86–1.21)
	Other	1
Ethnicity	White	0.81 (0.60–1.09)
	Other	1
Born in the UK	Yes	1.33 (1.01–1.74)*
	No	1
Living alone	No	1
	Yes	1.13 (0.94–1.35)
Children in the household	No	1
	Yes	0.80 (0.67–0.95)*
Depression (PHQ‐9) caseness	No	1
	Yes	0.83 (0.67–1.03)
Anxiety (GAD‐7) caseness	No	1
	Yes	0.98 (0.77–1.24)
COVID‐19 PTSD caseness	No	1
	Yes	1.02 (0.82–1.27)
Mental health treatment	Current/past mental health treatment	0.75 (0.64–0.88)***
	Other	1
Loneliness caseness	No	1
	Yes	0.99 (0.84–1.16)
Neuroticism		1.02 (0.97–1.06)
Hopefulness		0.95 (0.91–0.99)*
Conspiracy mentality		0.98 (0.98–0.99)***
Paranoia		0.99 (0.97–1.01)
COVID‐19 anxiety		1.00 (0.99–1.00)

^a^ Participants classified as ‘Other gender’ not included due to low cell count.

*p < 0.05, **p < 0.01, ***p < 0.001.

Importantly, probable diagnoses of major depression, generalised anxiety disorder, or COVID‐19 related PTSD at the previous wave (C19PRC‐UKW4, conducted during November–December 2020) did not predict attrition at C19PRC‐UKW5; however, adults reporting a history of current or past mental health treatment at C19PRC‐UKW4 were at lower odds of participating in C19PRC‐UKW5 (OR = 0.75; 95%CI 0.64–0.88). Of the psychological characteristics analysed, only lower levels of hopefulness (OR = 0.95; 95%CI = 0.91–0.99) and conspiracy mentality (OR = 0.98; 95%CI 0.98–0.99) were associated with attrition between these two survey waves, though effect sizes were relatively small.

### 3.4 Attrition analysis for baseline entrants only by C19PRC‐UKW5

By this fifth wave, 1162 of the 2025 adults recruited at baseline (57.4%) were re‐interviewed. Almost six‐in‐ten (*N* = 677; 58.3%) of those who were re‐interviewed had participated in all four previous C19PRC Study waves; three‐in‐ten (*N* = 351; 30.2%) participated in any three previous waves, one‐in‐ten (*N* = 117; 10.1%) in any two previous waves, and a small number (*N* = 17; 1.5%) returned having only participated at baseline.

Table 5 compares the socio‐demographic characteristics of reinterviewed baseline respondents at C19PRC‐UKW5 (second column) to the characteristics of all respondents recruited at baseline (*N* = 2025; first column). Attrition occurred in higher proportions among baseline respondents who were younger (particularly those aged 18–24 years), female, living in cities, of non‐White ethnicity, had children living in the household, were born or raised outside the UK, or had probable depression, anxiety, or COVID‐19 PTSD at baseline. As presented in the third column of Table 4, the raking procedure successfully re‐balanced the characteristics of responders at this fifth wave (*N* = 1162) to the baseline proportions for gender and age (exact rebalance), household income (within 1.1%), household composition and urbanicity (exact rebalance), ethnicity (within 0.1%), and status relating to being born or raised in the UK (within 1.5%). Applying this weight for all analyses of the C19PRC‐UKW5 survey data completed by this longitudinal panel of adults recruited and followed from baseline is recommended to account for attrition over survey waves on core study outcomes.

**TABLE 5:** Outcome of the raking weighting procedure conducted at C19PRC‐UKW5, March–April 2021, for the longitudinal panel recruited at baseline and followed‐up at this survey wave (*N* = 1162)

	C19PRC‐UKW1 (Mar–Apr 2020)	C19PRC‐UKW5 recontacts* *N* (unweighted)/% (weighted)
Age		
18–24	246 (12.1%)	57 (12.1%)
25–34	380 (18.8%)	172 (18.8%)
35–44	353 (17.4%)	185 (17.4%)
45–54	410 (20.2%)	267 (20.2%)
55–64	349 (17.2%)	258 (17.2%)
65+	287 (14.2%)	223 (14.2%)
Gender^a^		
Male	972 (48.0%)	601 (48.0%)
Female	1053 (52.0%)	561 (52.0%)
Income		
£0–300 per week	410 (20.2%)	237 (19.1%)
£301–490 per week	410 (20.2%)	210 (19.9%)
£491–740 per week	385 (19.0%)	226 (19.9%)
£741‐1111 per week	410 (20.2%)	239 (20.2%)
£1112 or more per week	410 (20.2%)	250 (20.9%)
Urbanicity		
City	498 (24.6%)	233 (24.6%)
Suburb/Town/Rural	1527 (75.4%)	929 (75.4%)
Ethnicity		
White	1848 (91.3%)	1081 (91.4%)
Non‐white	177 (8.7%)	81 (8.6%)
Household composition		
Children in household	592 (29.2%)	290 (29.2%)
No children in household	1433 (70.8%)	872 (70.8%)
Born or raised in UK		
Yes	1891 (93.4%)	1103 (94.9%)
No	134 (6.6%)	59 (5.1%)
Depression caseness		
Yes	448 (22.1%)	202 (20.0%)
No	1577 (77.9%)	960 (80.0%)
Anxiety caseness		
Yes	438 (21.6%)	205 (20.6%)
No	1587 (78.4%)	957 (79.4%)
PTSD caseness		
Yes	340 (16.8%)	160 (16.2%)
No	1685 (83.2%)	1002 (83.8%)

^a^ Other gender categories combined with female for purposes of weighting.

*Recontacted from C19PRC‐UKW1 only.

The characteristics of the C19PRC‐UKW5 longitudinal panel (*N* = 1162) by gender and age group were examined (see Table S3). Similar to the cross‐sectional sample, higher proportions of females in the youngest age bands were evident (71.4% of 18–24 year olds and 72.0% of 25–34 years older were female), whereas males dominated the older age groups (57.2% of participants in the 55–64 years and 65+ year age groups were male).

## 4 DISCUSSION

The C19PRC Study is a dynamic, longitudinal survey, which contains a broad array of socio‐political, economic, and mental health measures that can be used to test a range of research questions relating to how the COVID‐19 pandemic has (and is) impacting the lives of ordinary citizens living in the UK. Consistent with our Consortium's ethos, we have documented the design and progress of the C19PRC Study up to the point of the first year post‐baseline, with the hope of stimulating awareness of the availability of this rich data resource and encouraging exploitation of the data for ongoing COVID‐19 related interdisciplinary research.

The main findings from our analyses can be summarised succinctly. First, re‐contact rates in the C19PRC Study are strong, as demonstrated by our ability at this fifth wave to re‐interview 61.5% of adults who participated in the previous wave (during November–December 2020), and 57.8% of all adults recruited at baseline (in March 2020). Second, attrition between the fourth and fifth waves (i.e., between winter 2020 and spring 2021) was largely predicted by socio‐demographic factors (i.e., younger age, lower household income, and having dependent children living in the household), experiences of treatment for mental health difficulties, and certain psychological characteristics (e.g., lower levels of hopefulness), but not experiences of common mental health conditions such as depression, anxiety, or posttraumatic stress respondents reported towards the end of 2020. Third, weighting procedures employed to account for attrition in the core longitudinal panel were successful in re‐balancing the sample with respect to characteristics of the baseline sample. And fourth, the C19PRC‐UKW5 cross‐sectional sample is large and diverse, and many new measures were introduced at this wave (e.g., prolonged grief disorder; perceived burdensomeness and thwarted belongingness; mania) which provides a unique opportunity to explore nuanced research questions relating to mental health experiences 1 year into the COVID‐19 pandemic in the UK.

Our Consortium has advocated previously that researchers conducting COVID‐19 related survey research should be transparent with respect to methodologies and recruitment practices for survey fieldwork conducted (largely online) during this time. Indeed, we have previously debated two core potential weaknesses in our study protocol, that is, the opt‐in, non‐probability‐based web panel of adults recruited according to pre‐determined quotas, and a reliance on a single mode of survey administration (internet‐based survey) (see McBride, Butter, Murphy, Shevlin, Hartman, Bennett, et al., 2021; McBride, Butter, Murphy, Shevlin, Hartman, Hyland, et al., 2021; McBride, Murphy, et al., 2021).

Here, we focus on four additional methodological issues that critics might highlight as additional potential weaknesses: (i) average retention rates; (ii) the non‐routine sample replenishment to account for attrition (conducted at the third and fourth waves only); (iii) the generation of weights for the longitudinal panel (followed from baseline) to adjust for attrition against quotas determined to recruit the baseline sample; and (iv) sample diversity/representativeness. We take the opportunity here to address each of these in turn.

The evidence indicates that retention rates over the five C19PRC survey waves compare favourably to other established panel studies in the UK, including those that have been repurposed to collect COVID‐19 specific survey data during this turbulent year. For example, by the fifth wave of the British Election Study in 2015 (the first wave was conducted in 2014), 58.5% of baseline respondents were retained (British Election Study, 2019). More recently, members of the Understanding Society (UKHLS) Wave 9 panel (*N* = ∼27K), which pre‐existed the pandemic and adopted probability‐based sampling methods and dual modes of survey administration (web and telephone), were invited to participate in a dedicated COVID‐19 survey. During April 2020 and March 2021, nine survey waves were administered to these participants and the proportion of respondents providing either full or partial web‐based interviews dropped from 65% to 46% during this time period (Understanding Society, 2021). Against these studies, we argue that the C19PRC Study competes well with respect to retention rates.

All longitudinal studies face challenges relating to attrition over time, and experts disagree as to whether attrition is more or less problematic when respondents are surveyed frequently (e.g., multiple times in a 1‐year period) compared to when longer gaps occur between contact (e.g., several years between survey waves) (Laurie, 2008). The rapid unfolding context of the pandemic necessitated frequent survey waves, although our findings suggest that this has not been unduly problematic with respect to attrition. Indeed, the C19PRC Study has successfully limited ‘complete’ loss‐to‐follow‐up over time by re‐contacting all participants at each survey wave with tailored and engaging invitation communications and inviting them to continue to participate. This ensures that the C19PRC Study is collecting data from participants when they do participate and, through the application of appropriate analytic methods (e.g., employing robust maximum likelihood estimation), these data can be used to address research questions of a longitudinal nature (see Shevlin et al., 2021). Moreover, consistent with other established and reputable panel studies (e.g., the American National Election Study), we have engaged in sample replenishment procedures. Funding constraints dictated that sample replenishment was only feasible at specific C19PRC survey waves, and a strategic decision was taken to undertake this process at the third and fourth waves only (July–August 2020 and November–December 2020), which is the mid‐point in the panel study (running between March 2020–November 2021). This process ensured that the cross‐sectional sample at these waves was sufficient to conduct meaningful analyses (*N* > 2K), but not so large as to impede continued follow‐up of all study participants moving forward in subsequent survey waves.

Our Consortium adopted the strategy of accounting for attrition in the longitudinal panel (recruited at baseline) by generating weights to re‐balance data for this sample re‐interviewed at post‐baseline waves to the socio‐demographic characteristics of all adults participating in the baseline sample. Given that the core C19PRC Study outcomes were mental health conditions, we felt it was a suitable approach to account for the fact that respondents with better health status tend to continue to participate in post‐baseline survey waves (Radler & Ryff, 2010). We recognise, however, that alternative approaches may be more suitable for specific types of quantitative analyses, and secondary users of the C19PRC Study data may wish to generate additional and/or alternative weights if using the data to address specific research questions.

Finally, whilst it has been demonstrated that the C19PRC Study cross‐sectional samples and the longitudinal panel are representative of the UK adult general population overall, the composition of the sample may not directly mirror the national population with respect to specific sub‐groups (e.g., distribution of gender across the age groups). Quota sampling methods at baseline was not designed to be interlocking across gender, age, and household income, however, and secondary users of the data should be cognisant of this. We also acknowledge that the recruitment from existing online marketing research panels likely excluded from the C19PRC Study specific sub‐groups of the population who may be particularly vulnerable to wide‐reaching effects of the pandemic (e.g., the homeless, those not connected to the internet, etc.).

In conclusion, however, the C19PRC Study data is a large, content‐rich, survey data resource. We strongly encourage secondary use of this survey data by researchers and stakeholders interested in addressing pertinent research questions which will contribute to the existing evidence base on the impact of the COVID‐19 pandemic on the lives of citizens of the UK now and into the future.

## CONFLICT OF INTEREST

All authors declare no conflict of interest.

## Supporting information

Supporting Information 1

Supporting Information 2

## Data Availability

The data and associated documentation related to C19PRC‐UKW1–C19PRC‐UKW6 are publicly available via the Open Science Framework (see https://osf.io/v2zur/files/).
