# Supplementary material for: Tracking the psychological and socio‐economic impact of the COVID‐19 pandemic in the UK: A methodological report from Wave 5 of the COVID‐19 Psychological Research Consortium (C19PRC) Study
Source: Int J Methods Psychiatr Res. 2022 Jun 27;31(4):e1928. doi: 10.1002/mpr.1928 (PMC9349513; doi:10.1002/mpr.1928)
Supplement: Supplementary file 1 — Supporting Information 1 [file MPR-31-e1928-s002.docx]

Supplementary Table S1 CHERRIES Checklist for the fifth wave of the COVID-19 Psychological Research Consortium (C19PRC) Study - C19PRC-UKW5 - March-April, 2021.

| **Item category** | **Item description** | **Description** |
| --- | --- | --- |
| Design | Study design | The COVID-19 Psychological Research Consortium (C19PRC) Study is an online, longitudinal study which was designed to measure, assess, and monitor the population’s psychological and social adjustment to the COVID-19 pandemic. At baseline (C19PRC-UKW1), quota sampling methods were employed to achieve a representative sample in terms of age, sex and household income. At Wave 5 (C19PRC-UKW5), all respondents who had previously participated in the survey were recontacted and invited to participate in the fifth wave. Participants were required to be an adult (aged 18+), able to read and write in English, and a resident of the UK. No other exclusion criteria were applied. |
| Ethics | Ethical approval | Ethical approval for the project was provided by the University of Sheffield (Reference number 033759). |
|  | Informed consent | As in previous waves, participants were informed that their data would be treated in confidence, that geolocating would be used to determine the area in which they lived (in conjunction with their residential postcode stem), and of their right to terminate participation at any time. Participants were also informed that some topics might be sensitive or distressing (e.g., self-harm/suicide content). Information about how their data would be stored and analysed by the research team was also provided. Participants were also informed that the survey would take approximately 30 minutes to complete and that they would be re-contacted at a later date to invite them to participate in subsequent survey waves. Participants provided informed electronic consent prior to completing the survey and were directed to contact the NHS website upon completion if they had any concerns about COVID-19, and emotional support services if they had been negatively impacted by any of the questions asked during the survey. |
|  | Data protection | Participants were informed that C19PRC data will be stored confidentially in line with GDPR. They were also informed that they would not be identifiable from any publications that arise from the survey and that their data would be shared with other researchers. When the study data was deposited with the UK Data Service and the OSF, location data was removed and replaced with relevant socioeconomic summary data (e.g., area-level deprivation and population density data). All other personal data was also removed. Versions of the survey data files with personal/identifiable information exist, but these files are restricted access only by the core investigating team. These files are held securely as password protected files on the Google/One Drive cloud systems of the investigating institutions. |
| Development and pre-testing | Development and testing | The questionnaire was developed by the C19PRC Study team, adapting earlier versions of the survey for C19PRC-UKW5. The survey was built in, and managed by, the survey company Qualtrics. Prior to full launch, multiple validation checks were conducted on the survey. First, the survey was piloted by several members of the C19PRC Study team before a ‘soft launch’ pilot was conducted to rectify sequencing/coding errors and omissions prior to the full launch. The soft launch also calculates the median survey completion time, providing an opportunity to tailor the content to ensure the median survey time does not exceed 30 minutes; this is important to minimise respondent burden and maximise participation over time. The C19PRC-UKW5 soft launch conducted using a new general population sample of (n=51) on 22 March 2021, and the median survey completion time was 19 mins 34 seconds. Participants in the soft launch are excluded from the final sample for the survey wave. |
| Recruitment process | Open vs closed survey | This was an open survey. Fieldwork for the C19PRC Study was conducted by the survey company Qualtrics. |
|  | Contact mode | Potential respondents were initially alerted to the C19PRC Study by Qualtrics in one of two ways: (1) they opted to enter studies they were eligible for by signing up to a panel platform; or (2) they received automatic notification through a partner router that alerted/directed them to studies for that they were eligible (either via email, SMS, and in-app notifications). Importantly, to avoid self-selection bias, survey invitations to eligible participants only provide general information and did not include specific details about the contents of the survey. At C19PRC-UKW5, Qualtrics re-contacted all adults who participated in any previous C19PRC survey waves via email, SMS, or in-app notifications and invited them to participate further in this survey, with invitations tailored to remind adults of their participation in a previous survey wave(s). Qualtrics' partners released invitations in batches and, after the initial invitation was received, respondents who had not completed the survey were sent two reminders to encourage them to participate. The first reminder was sent approximately 36–48 h after the initial survey invite, with the second reminder sent another 36–48 h after this first reminder. |
|  | Advertising the survey | The survey was not advertised; as above, Qualtrics distributed the survey link via their partners. |
| Survey administration | Web/email | Those who chose to participate followed a link to a secure website and completed all surveys online. The invite link was active for a participant until a quota they would have qualified for was reached but after this time, previously eligible respondents were prevented from taking part. Responses were automatically collected and stored on the Qualtrics survey platform. |
|  | Context | Qualtrics partners with over 20 online sample providers to supply a network of diverse, quality respondents to their worldwide client base and, to date, has completed more than 15,000 projects across 2,500 universities worldwide. As an aggregator of panels, Qualtrics provides the online platform to securely house data and leverages partners to connect with respondents. Qualtrics recruits study participants from traditional, actively managed, double-opt-in market research panels, that are used for corporate and academic market research only. |
|  | Mandatory/voluntary | Voluntary. |
|  | Incentives | Panel members routinely receive an incentive for survey participation based on the length of the survey, their specific panel list profile, and target acquisition difficulty, among other factors. The specific type of reward varies and may include cash, air miles, gift cards, redeemable points, charitable donations, sweepstakes entrance, or vouchers. |
|  | Time/date | Responses were collected between 24 March – 20 April 2021. |
|  | Item randomisation | No randomisation of items was used. |
|  | Adaptive questioning | Adaptive questioning (e.g. skip logic, display logic) was used in the survey to reduce the number and complexity of questions. For example, items in the ‘parenting’ module of the survey were only presented to those who reported being a parent of a child under the age of 18 or adults who reported having children under 18 living in their home. |
|  | Number of items | There was an average of 5 items per page. Due to the adaptive nature of the survey, not all respondents completed all items. |
|  | Number of screens | The full survey was distributed over approximately 81 screens/pages. Due to the adaptive nature of the survey, not all respondents completed all pages. |
|  | Completeness checks | Respondents were prompted to complete all mandatory items before preceding to the next page of the survey. The vast majority of survey items were coded as mandatory (using the ‘force response’ option in Qualtrics) and where necessary, “not applicable”, “don’t know”, “none of the above”, “not sure” or “prefer not to say” options were provided. Items relating to particularly sensitive issues (self-harm, suicide and traumatic childhood experiences) were not mandatory and these section, or particular items within these sections, could be skipped by the respondent. |
|  | Review step | Participants could use a ‘back’ button to review and change their answers. |
| Response rates | Unique site visitor | Determination of unique visitors was handled by the panel provider Qualtrics. Qualtrics’ partners allocate each panel member a unique ID number when they sign up to their panel, which is automatically captured as part of their survey response. As described below, cookies and IP addresses were also used to prevent multiple entries from the same individual. |
|  | View rate | Not applicable. Respondents were invited to take part through Qualtrics’ panels. |
|  | Participation rate | Across Phases 1 and 2, 4949 adults were recontacted (having completed one or more earlier waves) and invited to take part at C19PRC-UKW5. Of these, 2657 were successfully recontacted (53.7% recontact rate). |
|  | Competition rate | Across Phases 1 and 2, 4949 adults were recontacted (having completed one or more earlier waves) and invited to take part at C19PRC-UKW5. Of these, 2520 successfully completed the survey (50.1% retention rate). |
| Preventing multiple entries from the same individual | Cookies used | Qualtrics used the ‘Ballot Box Stuffing’ feature to track respondents’ cookies to identify whether they had entered and/or completed the survey before. |
|  | IP check | Qualtrics worked with a specialist data quality team to identify any potential duplicate respondents in the survey. This ‘scrubbing partner’ reviewed the dataset and identified duplicates based on their response ID, IP address and/or response patterns. Any cases identified through this manner were removed from the dataset, keeping the first iteration of each duplicate. The research team additionally screened for any duplicates that had previously been missed and removed those also. |
|  | Log file analysis | As described above, Qualtrics’ ‘scrubbing partner’ used several techniques to identify multiple entries by the same respondent. |
|  | Registration | A login was not used. Eligible participants gained entry to the survey through a link which was made available to them via email, SMS or in-app notifications. Duplicate entries were handled as described above. |
| Analysis | Handling on incomplete questionnaires | Only completed questionnaires were included in the final dataset. |
|  | Questionnaires with atypical timestamp | Each participant must achieve ‘legitimate respondent status’ upon entry into the survey. This means that the respondent must spend a minimum amount of time completing the survey (i.e. half the median soft launch completion time for that wave) the first time they participate. Respondents who do not achieve this status are flagged as ‘speeders’ and removed from the study. Since, at C19PRCUK-W5, no new respondents were recruited into the study, all participants were considered ‘legitimate responders’, based on their competition times at their entry wave and therefore, no cases were removed for completing the survey too quickly. The median completion time for the C19PRC-UKW5 survey was 31 minutes, 28 seconds. |
|  | Statistical correction | Post-stratification survey weighting was used for the longitudinal panel, using the ‘raking’ method. In practice, this means the baseline sampling quotas for age, gender, and household income (nationally representative), as well as the baseline proportions achieved for ethnicity, urbanicity, household composition, and being born or raised in the UK, were imposed on the sample of respondents returning from baseline at C19PRC-UKW5, and the raking algorithm was conducted to produce, and iteratively adjust, a weight value for each case in the sample until the sample distribution aligned with the population distribution for the chosen characteristics. The maximum weight allowed was 5.00, and in the final panel, weights ranged from 0.72 - 3.00. |

This checklist is based on the Checklist for Reporting Results of Internet E-Surveys (CHERRIES) (Eysenbach, 2004).

Eysenbach, G. (2004). Improving the Quality of Web Surveys: The Checklist for Reporting Results of Internet E-Surveys (CHERRIES). *Journal of Medical Internet Research, 6*(3), e34 [correction published in Journal of Medical Internet Research (2012), 14(1), e8.].

Supplementary Table S2 Socio-demographic characteristics and prevalence of mental health disorders of the C19PRC-UKW5 cross-sectional sample (N=2520) (March-April, 2021), by age and gender

| **Respondent characteristics (C19PRC-UKW5)** | | **Gender** | | | | **Age group** | | | | | | | | | | | | | | | **Total** |
| --- | --- | --- | --- | --- | --- | --- | --- | --- | --- | --- | --- | --- | --- | --- | --- | --- | --- | --- | --- | --- | --- |
|  |  | **N(%)** | | | | **N(%)** | | | | | | | | | | | | | | | **N(%)** |
|  | | **Male** | | **Female** | | **18-24** | **25-34** | | | **35-44** | | | **45-54** | | | **55-64** | | | **65+** | |  |
| **Socio-demographic** | |  | |  | |  |  | | |  | | |  | | |  | | |  | |  |
| Gender^1^ | Male | - | | - | | 36 (37.1) | 87 (26.0) | | | 194 (46.3) | | | 281 (54.5) | | | 328 (55.3) | | | 341 (60.9) | | 1267 (50.3) |
|  | Female | - | | - | | 60 (61.9) | 245 (73.1) | | | 225 (53.7) | | | 234 (45.3) | | | 264 (44.5) | | | 218 (38.9) | | 1246 (49.4) |
| Age group (years) | 18-24 years | 36 (2.8) | | 60 (4.8) | | - | - | | | - | | | - | | | - | | | - | | 97 (3.8) |
|  | 25-34 years | 87 (6.9) | | 245 (19.7) | | - | - | | | - | | | - | | | - | | | - | | 335 (13.3) |
|  | 35-44 years | 194 (15.3) | | 225 (18.1) | | - | - | | | - | | | - | | | - | | | - | | 419 (16.6) |
|  | 45-54 years | 281(22.2) | | 234 (18.8) | | - | - | | | - | | | - | | | - | | | - | | 516 (20.5) |
|  | 55-64 years | 328 (25.9) | | 264 (21.2) | | - | - | | | - | | | - | | | - | | | - | | 593 (23.5) |
|  | 65+ years | 341 (26.9) | | 218 (17.5) | | - | - | | | - | | | - | | | - | | | - | | 560 (22.2) |
| 2019 household income | ≤£15,490 | 215 (17.0) | | 263 (21.1) | | 17 (17.5) | 43 (12.8) | | | 63 (15.0) | | | 106 (20.5) | | | 155 (26.1) | | | 97 (17.3) | | 481 (19.1) |
|  | £15,491-£25,340 | 223 (17.6) | | 241 (19.3) | | 20 (20.6) | 99 (29.6) | | | 79 (18.9) | | | 66 (12.8) | | | 83 (14.0) | | | 118 (21.1) | | 465 (18.5) |
|  | £25,341-£38,740 | 276 (21.8) | | 285 (22.9) | | 31 (32.0) | 92 (27.5) | | | 91 (21.7) | | | 91 (17.6) | | | 100 (16.9) | | | 158 (28.2) | | 563 (22.3) |
|  | £38,741-£57,903 | 268 (21.2) | | 249 (20.0) | | 15 (15.5) | 61 (18.2) | | | 99 (23.6) | | | 112 (21.7) | | | 125 (21.1) | | | 106 (18.9) | | 518 (20.6) |
|  | ≥£57,931 | 285 (22.5) | | 208 (16.7) | | 14 (14.4) | 40 (11.9) | | | 87 (20.8) | | | 141 (27.3) | | | 130 (21.9) | | | 81 (14.5) | | 493 (19.6) |
| Economic activity | Employed (incl. full or part-time, self-employed, and furloughed) | 739 (58.3) | | 734 (58.9) | | 64 (66.0) | 264 (78.8) | | | 345 (82.3) | | | 394 (76.4) | | | 331 (55.8) | | | 77 (13.8) | | 1475 (58.5) |
|  | Other | 528 (41.7) | | 512 (41.1) | | 33 (34.0) | 71 (21.2) | | | 74 (17.7) | | | 122 (23.6) | | | 262 (44.2) | | | 483 (86.3) | | 1045 (41.5) |
| Relationship status | Married | 694 (54.8) | | 567 (45.5) | | 3 (3.1) | 102 (30.4) | | | 199 (47.5) | | | 250 (48.4) | | | 345 (58.2) | | | 363 (64.8) | | 1262 (50.1) |
|  | Civil partnership | 8 (0.6) | | 1 (0.1) | | - | - | | | 1 (0.2) | | | 1 (0.2) | | | 4 (0.7) | | | 3 (0.5) | | 9 (0.4) |
|  | Cohabiting | 115 (9.1) | | 179 (14.4) | | 7 (7.2) | 77 (23.0) | | | 66 (15.3) | | | 82 (15.9) | | | 46 (7.8) | | | 17 (3.0) | | 295 (11.7) |
|  | Committed relationship | 77 (6.1) | | 99 (7.9) | | 28 (28.9) | 44 (13.1) | | | 20 (4.8) | | | 30 (5.8) | | | 31 (5.2) | | | 24 (4.3) | | 177 (7.0) |
|  | Single | 373 (29.4) | | 400 (32.1) | | 59 (60.8) | 112 (33.4) | | | 133 (31.7) | | | 153 (29.7) | | | 167 (28.2) | | | 153 (27.3) | | 777 (30.8) |
| Sexuality | Heterosexual | 1116 (88.1) | | 1170 (93.9) | | 80 (82.5) | 289 (86.3) | | | 363 (86.6) | | | 456 (88.4) | | | 557 (93.9) | | | 543 (97.0) | | 2288 (90.8) |
|  | Gay/lesbian/homosexual | 95 (7.5) | | 18 (1.4) | | 5 (5.2) | 19 (5.7) | | | 32 (7.6) | | | 30 (5.8) | | | 20 (3.4) | | | 9 (1.6) | | 115 (4.6) |
|  | Bisexual | 38 (3.0) | | 37 (3.0) | | 7 (7.2) | 19 (5.7) | | | 12 (2.9) | | | 21 (4.1) | | | 11 (1.9) | | | 5 (0.9) | | 75 (3.0) |
|  | Other/prefer not to say | 18 (1.4) | | 21 (1.7) | | 5 (5.2) | 8 (2.4) | | | 12 (2.9) | | | 9 (1.7) | | | 5 (0.8) | | | 3 (0.5) | | 42 (1.6) |
| Household characteristics^2^ | Single adult household (i.e., living alone) | 328 (25.9) | | 283 (22.7) | | 17 (17.5) | 63 (18.8) | | | 98 (23.4) | | | 121 (23.4) | | | 161 (27.2) | | | 154 (27.5) | | 614 (24.4) |
|  | Other | 939 (74.1) | | 963 (77.3) | | 80 (82.5) | 272 (81.2) | | | 321 (76.6) | | | 395 (76.6) | | | 432 (72.8) | | | 406 (72.5) | | 1906 (75.6) |
|  | Children under 18 years living in household | 233 (18.4) | | 300 (24.1) | | 8 (8.2) | 112 (33.4) | | | 193 (46.1) | | | 158 (30.6) | | | 47 (7.9) | | | 15 (2.7) | | 533 (21.2) |
|  | Other | 1034 (81.6) | | 946 (75.9) | | 89 (91.8) | 223 (66.6) | | | 226 (53.9) | | | 358 (69.4) | | | 546 (92.1) | | | 545 (97.3) | | 1987 (78.8) |
| Urbanicity^2^ | Suburb/Town/Rural | 1025 (80.9) | | 1011 (81.1) | | 71 (73.2) | 245 (73.1) | | | 298 (71.1) | | | 408 (79.1) | | | 510 (86.0) | | | 508 (90.7) | | 2040 (81.0) |
|  | City | 242 (19.1) | | 235 (18.9) | | 26 (26.8) | 90 (26.9) | | | 121 (28.9) | | | 108 (20.9) | | | 83 (14.0) | | | 52 (9.3) | | 480 (19.0) |
| Country of residence | England | 710 (56.0) | | 720 (57.8) | | 85 (87.6) | 236 (70.4) | | | 268 (64.0) | | | 312 (60.5) | | | 282 (47.6) | | | 250 (44.6) | | 1433 (56.9) |
|  | Wales | 203 (16.0) | | 189 (15.2) | | - | 32 (9.6) | | | 46 (11.0) | | | 66 (12.8) | | | 125 (21.1) | | | 124 (22.1) | | 393 (15.6) |
|  | Scotland | 212 (16.7) | | 219 (17.6) | | 8 (8.2) | 46 (13.7) | | | 69 (16.5) | | | 82 (15.9) | | | 105 (17.7) | | | 122 (21.8) | | 432 (17.1) |
|  | Northern Ireland | 142 (11.2) | | 118 (9.5) | | 4 (4.1) | 21 (6.3) | | | 36 (8.6) | | | 56 (10.9) | | | 81 (13.7) | | | 64 (11.4) | | 262 (10.4) |
| **Mental health conditions and treatment** | | |  | |  | | | |  | | |  | | | | |  | | |  | |
| Depression (PHQ-9) | Caseness met | 223 (17.6) | | 318 (25.5) | | 39 (40.2) | 115 (34.3) | | | 108 (25.8) | | | 115 (22.3) | | | 112 (18.9) | | | 56 (10.0) | | 545 (21.6) |
|  | Not met | 1044 (82.4) | | 928 (74.5) | | 58 (59.8) | 220 (65.7) | | | 311 (74.2) | | | 401 (77.7) | | | 481 (81.1) | | | 504 (90.0) | | 1975 (78.4) |
| Anxiety (GAD-7) | Caseness met | 172 (13.6) | | 250 (20.1) | | 30 (30.9) | 99 (29.6) | | | 91 (21.7) | | | 94 (18.2) | | | 77 (13.0) | | | 34 (6.1) | | 425 (16.9) |
|  | Not met | 1095 (86.4) | | 996 (79.9) | | 67 (69.1) | 236 (70.4) | | | 328 (78.3) | | | 422 (81.8) | | | 516 (87.0) | | | 526 (93.9) | | 2095 (83.1) |
| COVID-19 PTSD | Caseness met | 144 (11.4) | | 154 (12.4) | | 32 (33.0) | 89 (26.6) | | | 77 (18.4) | | | 58 (11.2) | | | 25 (4.2) | | | 18 (3.2) | | 299 (11.9) |
|  | Not met | 1123 (88.6) | | 1092 (87.6) | | 65 (67.0) | 246 (73.4) | | | 342 (81.6) | | | 458 (88.8) | | | 568 (95.8) | | | 542 (96.8) | | 2221 (88.1) |
| Treatment history | Never received | 953 (75.2) | | 836 (67.1) | | 59 (60.8) | 204 (60.9) | | | 275 (65.6) | | | 358 (69.4) | | | 426 (71.8) | | | 469 (83.3) | | 1791 (71.1) |
|  | Received in the past | 192 (15.2) | | 245 (19.7) | | 16 (16.5) | 61 (18.2) | | | 77 (18.4) | | | 99 (19.2) | | | 115 (19.4) | | | 71 (12.7) | | 439 (17.4) |
|  | Currently receiving | 64 (5.1) | | 108 (8.7) | | 12 (12.4) | 36 (10.7) | | | 39 (9.3) | | | 42 (8.1) | | | 34 (5.7) | | | 9 (1.6) | | 172 (6.9) |
|  | Other | 58 (4.6) | | 57 (4.6) | | 10 (10.3) | 34 (10.1) | | | 28 (6.7) | | | 17 (3.3) | | | 18 (0.7) | | | 11 (0.4) | | 118 (4.6) |
| **COVID-19 related experiences and perspectives** | | | | | |  | |  | | |  | | |  |  | | |  | | |  |
| Self-isolated during pandemic | Yes | 290 (22.9) | | 320 (25.7) | | 25 (25.8) | 103 (30.7) | | | 114 (27.2) | | | 123 (23.8) | | | 129 (21.8) | | | 119 (21.3) | | 613 (24.3) |
|  | No | 977 (77.1) | | 926 (74.3) | | 72 (74.2) | 232 (69.3) | | | 305 (72.8) | | | 393 (76.3) | | | 464 (78.2) | | | 441 (78.8) | | 1907 (75.5) |
| Had COVID-19 | Yes | 92 (7.3) | | 98 (7.9) | | 8 (8.2) | 43 (12.8) | | | 37 (8.8) | | | 45 (8.7) | | | 38 (6.4) | | | 19 (3.4) | | 190 (7.5) |
|  | No | 1097 (86.6) | | 1061 (85.2) | | 84 (86.6) | 270 (80.6) | | | 346 (82.6) | | | 432 (83.7) | | | 511 (86.2) | | | 521 (93.0) | | 2164 (85.9) |
|  | Not sure | 78 (6.2) | | 87 (7.0) | | 5 (5.2) | 22 (6.6) | | | 36 (8.6) | | | 39 (7.6) | | | 44 (7.4) | | | 20 (3.6) | | 166 (6.6) |
| Vaccinated | Yes | 894 (70.6) | | 731(58.7) | | 24 (24.7) | 97 (29.0) | | | 159 (37.9) | | | 303 (58.7) | | | 505 (85.2) | | | 539 (96.3) | | 1627 (64.6) |
|  | No | 373 (29.4) | | 515 (41.3) | | 73 (75.3) | 238 (71.0) | | | 260 (62.1) | | | 213 (41.3) | | | 88 (14.8) | | | 21 (3.8) | | 893 (35.4) |
| Worst of pandemic | Behind us | 825 (65.1) | | 773 (62.0) | | 62 (63.9) | 201 (60.0) | | | 246 (58.7) | | | 335 (64.9) | | | 379 (63.9) | | | 378 (67.5) | | 1601 (63.5) |
|  | Happening now | 298 (23.5) | | 348 (27.9) | | 28 (28.9) | 101 (30.1) | | | 133 (31.7) | | | 124 (24.0) | | | 140 (23.6) | | | 122 (21.8) | | 648 (25.7) |
|  | Ahead of us | 144 (11.4) | | 125 (10.0) | | 7 (7.2) | 33 (9.9) | | | 40 (9.5) | | | 57 (11.0) | | | 74 (12.5) | | | 60 (10.7) | | 271 (10.8) |

Note. ^1 ‘^Other’ gender category not included due to low cell count (n=7); ^2^ Household characteristics ‘Living alone’ and Urbanicity were not measured at C19PRC-UKW5, so this information comes from the last wave the participant completed.

Supplementary Table S3 Socio-demographic characteristics and prevalence of mental health disorders of the C19PRC-UKW5 longitudinal panel from baseline (N=1162) (March-April, 2021), by age and gender

| **Respondent characteristics (C19PRC-UKW5)** | | **Gender** | | **Age group** | | | | | | **Total** |
| --- | --- | --- | --- | --- | --- | --- | --- | --- | --- | --- |
|  |  | **N(%)^1^** | | **N(%)^1^** | | | | | | **N(%)^1^** |
|  | | **Male** | **Female** | **18-24** | **25-34** | **35-44** | **45-54** | **55-64** | **65+** |  |
| **Socio-demographic** | |  |  |  |  |  |  |  |  |  |
| Gender^2^ | Male | - | - | 12 (28.4) | 44 (27.1) | 96 (49.3) | 159 (59.8) | 144 (54.6) | 139 (56.6) | 594 (47.5) |
|  | Female | - | - | 30 (71.6) | 113 (72.9) | 95 (50.7) | 105 (39.7) | 119 (45.4) | 104 (42.9) | 566 (52.3) |
| Age group (years) | 18-24 years | 12 (5.2) | 30 (12.0) | - | - | - | - | - | - | 42 (3.6) |
|  | 25-34 years | 44 (10.7) | 113 (26.2) | - | - | - | - | - | - | 157 (13.5) |
|  | 35-44 years | 96 (19.2) | 95 (17.9) | - | - | - | - | - | - | 191 (16.4) |
|  | 45-54 years | 159 (25.9) | 105 (15.6) | - | - | - | - | - | - | 265 (22.8) |
|  | 55-64 years | 144 (20.4) | 119 (15.5) | - | - | - | - | - | - | 263 (22.6) |
|  | 65+ years | 139 (18.6) | 104 (12.8) | - | - | - | - | - | - | 244 (21.0) |
| 2019 household income | ≤£15,490 | 106 (17.6) | 128 (18.9) | 7 (16.7) | 11 (6.4) | 30 (15.0) | 54 (20.2) | 85 (31.9) | 48 (19.9) | 235 (18.4) |
|  | £15,491-£25,340 | 103 (18.3) | 79 (15.6) | 6 (14.7) | 54 (33.0) | 31 (16.4) | 35 (13.0) | 19 (7.2) | 37 (14.9) | 182 (16.8) |
|  | £25,341-£38,740 | 108 (18.7) | 108 (20.9) | 13 (30.4) | 41 (27.1) | 35 (18.7) | 35 (13.0) | 28 (10.6) | 65 (26.5) | 217 (19.9) |
|  | £38,741-£57,903 | 125 (20.8) | 129 (23.4) | 8 (19.6) | 32 (21.1) | 46 (24.3) | 60 (23.1) | 56 (21.7) | 52 (21.0) | 254 (22.1) |
|  | ≥£57,931 | 152 (24.6) | 122 (21.2) | 8 (18.6) | 19 (12.4) | 49 (25.7) | 81 (30.7) | 75 (28.5) | 42 (17.7) | 274 (22.8) |
| Economic activity | Employed (incl. full or part-time, self-employed, and furloughed) | 376 (68.0) | 332 (64.1) | 32 (77.7) | 131 (83.5) | 156 (81.9) | 206 (77.7) | 147 (56.5) | 37 (14.9) | 709 (66.0) |
|  | Other | 218 (32.0) | 234 (35.9) | 10 (22.3) | 26 (16.5) | 35 (18.1) | 59 (22.3) | 116 (43.5) | 207 (85.1) | 453 (34.0) |
| Relationship status | Married | 322 (50.9) | 261 (41.0) | - | 52 (32.4) | 88 (47.2) | 133 (50.8) | 157 (60.2) | 153 (62.4) | 583 (45.7) |
|  | Civil partnership | 5 (0.7) | - | - | - | 1 (0.5) | - | 2 (0.5) | 2 (0.6) | 5 (0.3) |
|  | Cohabiting | 58 (10.5) | 87 (16.1) | 3 (6.8) | 34 (21.9) | 36 (18.5) | 46 (17.2) | 18 (6.8) | 8 (3.3) | 145 (13.4) |
|  | Committed relationship | 35 (6.3) | 48 (12.7) | 17 (40.8) | 20 (13.7) | 12 (6.5) | 10 (3.8) | 12 (4.4) | 12 (5.0) | 83 (9.7) |
|  | Single | 174 (31.5) | 170 (30.3) | 22 (52.4) | 51 (32.0) | 54 (27.3) | 76 (28.2) | 74 (28.2) | 69 (28.7) | 346 (31.0) |
| Sexuality | Heterosexual | 526 (88.1) | 530 (92.3) | 36 (87.3) | 143 (90.4) | 162 (85.1) | 232 (87.4) | 247 (93.7) | 237 (96.7) | 1057 (90.1) |
|  | Gay/lesbian/homosexual | 43 (8.0) | 10 (1.8) | 1 (2.0) | 7 (5.0) | 15 (7.4) | 18 (6.7) | 8 (3.4) | 5 (2.2) | 54 (4.8) |
|  | Bisexual | 16 (2.5) | 16 ( 3.9) | 4 (8.8) | 4 (2.8) | 6 (3.3) | 13 (5.0) | 4 (1.4) | 1 (0.5) | 32 (3.3) |
|  | Other/prefer not to say | 9 (1.4) | 10 (2.0) | 1 (2.0) | 3 (1.8) | 8 (4.2) | 2 (0.8) | 4 (1.4) | 1 (0.5) | 19 (1.8) |
| Household characteristics^3^ | Single adult household (i.e., living alone) | 153 (24.8) | 124 (19.4) | 2 (4.9) | 31 (18.9) | 45 (22.8) | 62 (22.7) | 71 (26.6) | 68 (28.2) | 279 (22.0) |
|  | Other | 441 (75.2) | 442 (80.6) | 40 (95.1) | 126 (81.1) | 146 (77.2) | 203 (77.3) | 192 (73.4) | 176 (71.8) | 883 (78.0) |
|  | Children under 18 years living in household | 124 (23.9) | 130 (25.8) | 3 (7.8) | 58 (36.2) | 85 (46.5) | 80 (32.8) | 24 (10.1) | 4 (1.7) | 254 (24.9) |
|  | Other | 470 (76.1) | 436 (74.2) | 39 (92.2) | 99 (63.8) | 106 (53.5) | 185 (67.2) | 239 (89.9) | 240 (98.3) | 908 (75.1) |
| Urbanicity^3^ | Suburb/Town/Rural | 484 (78.1) | 453 (75.0) | 32 (73.5) | 107 (64.7) | 135 (67.9) | 215 (79.0) | 227 (84.5) | 222 (89.6) | 938 (76.4) |
|  | City | 110 (21.9) | 113 (25.0) | 10 (26.5) | 50 (35.3) | 56 (32.1) | 50 (21.0) | 36 (15.5) | 22 (10.4) | 224 (23.6) |
| Country of residence | England | 498 (84.1) | 488 (87.3) | 38 (90.2) | 139 (89.9) | 164 (85.6) | 220 (83.2) | 219 (83.1) | 207 (85.1) | 987 (85.8) |
|  | Wales | 22 (3.3) | 22 (3.3) | - | 5 (2.8) | 4 (1.9) | 11 (4.2) | 10 (3.9) | 15 (6.1) | 45 (3.4) |
|  | Scotland | 60 (10.3) | 45 (7.7) | 4 (9.8) | 11 (6.5) | 14 (7.9) | 27 (10.1) | 30 (11.6) | 19 (7.7) | 105 (8.9) |
|  | Northern Ireland | 14 (2.4) | 11 (1.6) | - | 2 (0.9) | 9 (4.7) | 7 (2.5) | 4 (1.4) | 3 (1.1) | 25 (2.0) |
| **Mental health conditions and treatment** | | |  |  |  |  |  |  |  |  |
| Depression (PHQ-9) | Caseness met | 100 (18.5) | 127 (24.8) | 15 (36.3) | 48 (30.3) | 45 (23.7) | 50 (18.9) | 49 (18.8) | 22 (9.3) | 229 (21.9) |
|  | Not met | 494 (81.5) | 439 (75.2) | 27 (63.7) | 109 (69.7) | 146 (76.3) | 215 (81.1) | 214 (81.2) | 222 (90.7) | 933 (78.1) |
| Anxiety (GAD-7) | Caseness met | 85 (16.1) | 95 (18.6) | 12 (28.2) | 42 (25.7) | 39 (20.8) | 41 (15.5) | 35 (13.5) | 12 (5.0) | 181 (17.5) |
|  | Not met | 509 (83.9) | 471 (81.4) | 30 (71.8) | 115 (74.3) | 152 (79.2) | 224 (84.5) | 228 (86.5) | 232 (95.0) | 981 (82.5) |
| COVID-19 PTSD | Caseness met | 73 (15.4) | 67 (14.6) | 11 (26.2) | 38 (24.8) | 47 (25.1) | 28 (10.5) | 9  (3.9) | 7 (2.8) | 140 (14.9) |
|  | Not met | 521 (84.6) | 499 (85.4) | 31 (73.8) | 119 (75.2) | 144 (74.9) | 237 (89.5) | 254 (96.1) | 237 (97.2) | 1022 (85.1) |
| Treatment history | Never received | 439 (72.5) | 384 (66.0) | 25 (59.2) | 101 (64.2) | 120 (62.5) | 190 (71.8) | 184 (69.7) | 203 (82.9) | 823 (68.9) |
|  | Received in the past | 94 (15.7) | 109 (20.1) | 9 (22.3) | 25 (17.0) | 40 (20.8) | 47 (17.6) | 53 (19.7) | 31 (12.7) | 205 (18.1) |
|  | Currently receiving | 32 (6.1) | 45 (8.1) | 5 (10.7) | 14 (8.3) | 19 (10.2) | 16 (5.9) | 18 (7.2) | 5 (2.2) | 77 (7.2) |
|  | Other | 29 (5.6) | 28 (5.9) | 3 (7.8) | 17 (10.6) | 12 (6.5) | 12 (4.6) | 8 (3.4) | 5 (2.2) | 57 (5.8) |
| **COVID-19 related experiences and perspectives** | | | |  |  |  |  |  |  |  |
| Self-isolated during pandemic | Yes | 133 (23.1) | 149 (27.0) | 9 (22.5) | 45 (28.9) | 61 (32.6) | 57 (21.8) | 60 (22.7) | 51 (20.9) | 283 (25.2) |
|  | No | 461 (76.9) | 417 (73.0) | 33 (77.5) | 112 (71.1) | 130 (67.4) | 208 (78.2) | 203 (77.3) | 193 (79.1) | 879 (74.8) |
| Had COVID-19 | Yes | 49 (9.0) | 49 (8.7) | 3 (6.9) | 20 (11.9) | 20 (10.7) | 25 (9.6) | 19 (6.8) | 11 (4.4) | 98 (8.7) |
|  | No | 514 (85.5) | 477 (84.4) | 38 (90.2) | 123 (78.9) | 160 (83.7) | 223 (83.7) | 226 (86.4) | 222 (91.2) | 992 (85.0) |
|  | Not sure | 31 (5.4) | 40 (6.9) | 1 (2.9) | 14 (9.2) | 11 (5.6) | 17 (6.7) | 18 (6.8) | 11 (4.4) | 72 (6.3) |
| Vaccinated | Yes | 414 (64.4) | 337 (51.5) | 9 (21.6) | 50 (33.0) | 71 (37.2) | 158 (58.8) | 229 (87.4) | 235 (96.2) | 752 (57.7) |
|  | No | 180 (35.6) | 229 (48.5) | 33 (78.4) | 107 (67.0) | 120 (62.8) | 107 (41.2) | 34 (12.6) | 9 (3.8) | 410 (42.3) |
| Worst of pandemic | Behind us | 403 (67.2) | 366 (64.0) | 26 (61.2) | 103 (65.3) | 116 (60.0) | 182 (68.9) | 169 (64.3) | 174 (71.3) | 770 (65.4) |
|  | Happening now | 127 (21.7) | 144 (26.5) | 11 (26.2) | 42 (26.9) | 55 (29.3) | 56 (21.0) | 61 (23.2) | 46 (18.8) | 271 (24.2) |
|  | Ahead of us | 64 (11.1) | 56 (9.5) | 5 (12.6) | 12 (7.8) | 20 (10.7) | 27 (10.1) | 33 (12.6) | 24 (9.9) | 121 (10.4) |

Note. ^1^ Unweighted N, weighted %; ^2 ‘^Other’ gender category not included due to low cell count (n=2); ^3^ Household characteristics ‘Living alone’ and Urbanicity were not measured at C19PRC-UKW5, so this information comes from the last wave the participant completed.
