# Supplementary material for: Tracking the psychological and socio‐economic impact of the COVID‐19 pandemic in the UK: A methodological report from Wave 5 of the COVID‐19 Psychological Research Consortium (C19PRC) Study
Source: Int J Methods Psychiatr Res. 2022 Jun 27;31(4):e1928. doi: 10.1002/mpr.1928 (PMC9349513; doi:10.1002/mpr.1928)
Supplement: Supplementary file 2 — Supporting Information 2 [file MPR-31-e1928-s001.docx]

**Supplementary Measures Material**

**2. Methods**

**2.2 Measures**

Where appropriate/relevant, details are presented about the use of standardised and validated measures in this wave. Details are also provided where established/existing measures have been modified for the purposes of collecting data during the pandemic. Unless otherwise stated, all other measures were derived by the C19PRC Study team for the purpose of collecting bespoke pandemic-specific data from survey respondents.

**2.2.1 Socio-demographic characteristics.** A detailed account of respondents’ socio-demographic history was collected: *age* (18-24 years; 25-34 years; 35-44 years; 45-54 years; 55-64 years; 65 years and over), *gender* (male, female, transgender, other, prefer not to say), *country of residence* (England, Scotland, Wales, Northern Ireland); *type of secondary education* (state comprehensive school; state grammar school; private day school; private boarding school; home-school; other – specify; did not attend secondary school; if boarding school selected, the respondent was asked to report the age when they first started attending); *religious identify* (Catholic; Protestant; Sunni; Shia; Jewish; Buddhist; Sikh; Hindu; Atheist; Agnostic; or other-specify); and *sexual orientation* (straight/heterosexual; gay/lesbian/homosexual; bisexual; other; prefer not to say).

*2.2.1.1 Relationship status*. Respondents were asking to report their current relationship status and their partner’s ethnicity, where applicable (the respondent’s legal/marital status and ethnicity were collected at previous waves). The following statement was presented: ‘*We’d like to ask you about your current relationship status at this point in time (how you are living now*): (1) single – not currently in a committed relationship but have previously been in a committed relationship; (2) single – never been in a committed relationship; (3) in a committed relationship but not living together; (4) cohabiting; (4) married; (5) in a civil partnership. For those with a previous relationship, they were asked to report on the status of the relationship: (1) divorced or were formally in a civil partnership which is now legally dissolved; (2) widowed or are the surviving partner from a civil partnership; (3) separated but marriage/civil partnership not yet legally dissolved; or (4) none of the above. Time (in years) since divorce or being widowed/survivor of a civil partnership was collected, where applicable. Respondents were asked to report on their partner’s ethnicity using the following categories: White British/Irish; White non-British/Irish; Indian; Pakistani; Chinese; Afro-Caribbean; African; Arab; Bangladeshi; Other Asian; Other -specify.

*2.2.1.2. Parental status and children living in the household.* Parental status was ascertained for all respondents, by providing the following statements (tick all that apply): (1) I do not have any children; (2) I have a child/children under 18 years or age, and he/she/they primarily live with me in my household; (3) I have a child/children under 18 years of age, but he/she/they primarily live elsewhere; (4) I have a child/children aged 18 years or over, and he/she/they primarily live with me in my household; (5) I have a child/children aged 18 years or over, but he/she/they primarily live elsewhere; and (6) someone else’s child/children under 18 years of age lives with me in my household.

All respondents were asked whether they or their partner were pregnant at the time of the survey and, if so, how many weeks? Respondents were also asked whether any members of their immediate family were pregnant at the time of the survey (yes/no response).

Respondents were also asked if they were planning to have a child/children in the future, with the following response options: Yes, No, Don’t know, I am unable to have children; Prefer not to say.

**2.2.2 Economic activity**

*2.2.2.1 Employment status.* Respondents were asked to indicate whether they were: employed full-time, employed part-time; self-employed (full-time), self-employed (part-time), been placed on the government ‘furlough’ scheme; unemployed, but looking for work; unemployed, looking after family or home; unemployed, long-term sick or disability; full-time student; or retired. Respondents who indicated they were unemployed, but looking for work were asked “*Are you unemployed as a consequence of the coronavirus pandemic?*” (Yes/No/Not sure).

*2.2.2.2 Keyworker status.* All respondents who indicated that they were not unemployed, a full-time student, or retired, were also asked to consider a definition of a ‘key worker’ (i.e. people whose jobs are vital to public health and safety during the coronavirus lockdown) and to determine whether their occupation was covered by any of the following categories (UK Cabinet Office/Department of Education, 2020): (1) Health and social care worker (e.g. all NHS staff including administrative and cleaning staff, care home workers); (2) Education and child care (e.g. nursery care workers and teachers); (3) Food and other necessary goods (e.g. staff involved in production, processing, distribution, sale and delivery of goods); (4) Key public services (e.g. postal workers, those required to run the justice system, religious staff, those responsible for managing the deceased and journalists providing public service broadcasting); (5) Local and national government (e.g. staff in administrative roles essential to the effective delivery of the COVID-19 response or delivering essential public services including payment of benefits); (6) Utility workers (e.g. staff needed to keep oil, gas, electricity, water and sewerage operations running, staff in the civil nuclear, chemical and telecom communications sectors); (7) Public safety and national security (e.g. police and support staff, Ministry of Defence civilian staff and armed forces personnel, fire and rescue staff, and workers responsible for border security, prisons, and probation); or (8) Transport (e.g. staff keeping air, water, road and rail passenger and freight transport modes operating).

*2.2.2.3 Underemployment* (Bell and Blanchflower (2021)). All respondents, regardless of current employment status, were administered the Bell-Blanchflower Unemployment Measure, which is a measure that assesses how well the labour force is being used in terms of skills, experience, and availability to work. Respondents were asked to consider “*Whatever your employment status, we would like now to ask you to respond to the following questions relating to hours of work*.” and answer the following questions (1) *how many hours a week do you typically work?; and (2) how many hours would you like to work?* An additional question was added *“how many hours a week did you typically work before the pandemic?* in order to capture changes in respondents’ typical working hours over the course of the pandemic. Responses to all three questions were recorded via an open text to insert a whole number.

**2.2.3 Household finances***.*

*2.2.3.1 Household income.* Self-estimated gross annual household income for 2019 was collected from all respondents using the following categories (which were used for quota sampling): (1) £0-£15,490; (2) £15,491-£25,340; (3) £25,341-£38,740; (4) £38,741-£57,930; and (5) £57,931 or more.

*2.2.3.2 Receipt of benefits*. A single-item was generated to collection information as to whether respondents were in receipt of benefits, as follows: “*Are you currently in receipt of any government benefits (not including child benefits and state pension)?*” (Yes/No response).

*2.2.3.3 Income changes during the pandemic.* Respondents were asked to “*Please estimate the percentage change (either increase or decrease) in their monthly household income compared to the average monthly income before the COVID-19*” with response recorded on a visual slider ranging from 100% Less to 100% More, centred (starting point) at No change. Respondents were also asked *“Are you making savings because of the COVID-19 pandemic (i.e., do you have more money at the end of the month)?”* and “*Are you using savings to help your household manage during the COVID-19 pandemic?”*, both scored using (1) yes, (2) no, or (3) don’t know. Respondents were also asked *“Has your overall debt increased or decreased this month due to COVID-19?”,* with responses scored on a 5-point Likert scale ranging from (1) increased a lot, to (5) decreased a lot; respondents were also able to indicate a response of ‘I do not have debt’. Respondents also self-reported their anxiety levels relating to household finances, as follows: “*On balance, how much are you worried about the way that your household finances have been affected by the coronavirus pandemic so far?*”. Responses were recorded on a 10-point Likert scale ranging from 1, not at all worried to 10, extremely worried. Finally, respondents were asked to consider “*Looking forwards, do you expect your financial security to* (1) get worse, (2) stay about the same, or (3) get better?”

*2.2.3.4 Future job loss/income security.* Respondents were asked to make predictions about their future income security: *“How likely do you think it is that, during the next ten years, you will suffer an unexpected loss of income, for example by losing a job you have or being unable to find a job when you need one?”* Responses were recorded on a visual slider ranging from 0 ‘Not at all likely’ to 100 ‘Extremely likely’, centred at 50 ‘Moderate likelihood’.

*2.2.3.5 Use of pandemic savings*. Individuals who had saved money as a result of the pandemic were asked to consider how they would use these savings: *Some people have saved money during the pandemic. If you have saved money, do you expect to spend some of it on major purchases such as a new car, a house renovation, house move or a special holiday.* Response options were (1) I have not saved money during the pandemic; (2) I have saved money but have no plans to spend it on big ticket items; or (3) I plan to spend some of the money I have saved on one or more major purchases. For those indicating an intention to spend savings on a major purchase, they were asked to report (ticking all that apply) if they plan to: (1) purchase a new car; (2) spend money on your house; (3) spend money moving house; (4) spend money on a special holiday; (5) spend money on another type of major purchase.

*2.2.3.6. Paying bills.* One question was adapted from the Eurobarometer Survey (Blanchflower & Clark, 2020) to assess adults’ difficulties with paying bills: “*During the last month, would you say you found it difficult to pay your bills?*, scored on a 4-point Likert scale ranging from (1) Not at all difficult to (4) Very difficult. The original question was asked with reference to the last 12 months, and scored on a 3-point Likert scale ranging from 1, never to 3, most of the time, but the stated adaptions were necessary to obtain recent data on respondents’ financial circumstances during the pandemic (i.e., to reflect that although a significant proportion of the population have experienced considerable economic hardship over the course of the pandemic, the income of many other households has largely been unaffected, or even improved, as a result of the pandemic).

*2.2.3.7. Food insecurity.* The eight-item Food Insecurity Experience Scale (FIES) (Food and Agricultural Organisation of the United Nation, 2016) was used to measure respondents’ experience of food insecurity, as follows: *“During the last 12 months, was there a time when you*: (1) You were worried you would run out of food because of a lack of money or other resources; (2) You were unable to eat healthy and nutritious food because of a lack of money or other resources; (3) You ate only a few kinds of foods because of a lack of money or other resources; (4) You had to skip a meal because there was not enough money or other resources to get food? (5) You ate less than you thought you should because of a lack of money or other resources; (6) Your household ran out of food because of a lack of money or other resources? (7) You were hungry but did not eat because there was not enough money or other resources for food? (8) You went without eating for a whole day because of a lack of money or other resources? (Yes/No responses).

*2.2.3.8. Social rank* (MacArthur Scale of Subjective Social Status; Adler, Epel, Castellazzo, and Ickovics (2000)). The MacArthur Scale of Subjective Social Status was developed to capture individuals’ sense of their place in the social ladder which accounts for multiple dimensions of socio-economic status (education, income, and occupation) and social position. Using a pictorial format, respondents were presented with a social ladder and asked to consider the following statement: “*Think of a ladder representing where people stand in the United Kingdom. At the top of the ladder are the people who are the best off – those who have the most money, the most education, and the most respected jobs. At the bottom are the people who are the worst off – those who have the least money, least education, and the least respected jobs or no job. The higher up you are on the ladder, the closer you are to the people at the very top; the lower you are, the closer you are to the people at the very bottom*”. *Please click the number below to show where you think you stand at this time in your life, relative to other people in the UK* (10-rungs ranging from 1, worst off, to 10, best off).

**2.2.4. Housing characteristics.**

*2.2.4.1. Physical properties of place of residence*. Respondents were firstly asked: “*Since you last completed this survey, has your living situation changed (e.g. you have moved house, someone has moved into or out of your home)?*” (Yes/No response). If yes, a series of questions were asked, as follows: Respondents provided information as to whether they lived alone or, if they lived with other people, how many adults lived with them. Furthermore, if respondents’ living situation had changed, they were asked also to provide information on:

(1) the type of property in which they live (an apartment, house or bungalow); (2) how many bedrooms are in the property (ranging from 0 bedrooms, a single-room dwelling, studio or flat to 5 or more); (3) their area of residence (living in a city, a suburb, a town or a rural area); and (4) housing tenure (response options: own outright; buying it with the help of a mortgage or loan; shared ownership; renting; living rent free; squatting; or other).

*2.2.4.2. Indoor and outdoor space.* Next, all respondents were asked a series of questions about the space within and outside their place of residence. Regarding outdoor space, respondents were asked whether their residence had a private garden, a shared garden, or a balcony (Yes/No response). Respondents were also report on the amount of private space they have in their home and the extent to which they agree with the following statements (scored on a 7-point Likert scale ranging from strongly disagree to strongly agree, not applicable option included): (1) If someone else is watching TV or playing music in the living area, there is somewhere you can go that is suitable and quiet; (2) The size and layout of your home does not allow you enough privacy; (3) Your children have a room with enough space in which they can play alone; (4) Your children can do their homework in private at a desk or table; (5) You can work in private at a desk or table; and (6) Your broadband coverage is suitable for your work needs; (7) Your broadband coverage is suitable for your social networking needs; and (8) Your broadband coverage is suitable for watching TV (streaming), gaming or listening to music.

*2.2.4.3 Belongingness in neighbourhood.* Three questions taken from the UK Community Liver Survey (Cabinet Office, 2015) were asked of respondents to assess their level of belongingness and connectedness to their neighbourhood generally and neighbours specifically, as follows: (1) *How strongly do you feel you belong to your immediate neighbourhood*? (scored on a 4-point scale from 1 ‘not at all’ to 4 ‘very much’; (2) *How comfortable would you be with asking a neighbour to keep a set of keys to your home for emergencies?* (scored on a 4-point scale ranging from 1 ‘very uncomfortable’ to 4 ‘very comfortable’); and (3) *How comfortable would you be asking a neighbour to collect a few shopping essentials for you, if you were ill and at home on your own*? (scored on a 4-point scale ranging from 1 ‘very uncomfortable’ to 4 ‘very comfortable’).

**2.2.5 COVID-19.**

The baseline wave (C19PRC-UKW1) was conducted in mid-March 2020 at the beginning of the pandemic when COVID-19 was a new virus, no existing measure was available to assess the general population’s knowledge, attitudes, and behaviours (KAB) of the virus. In order to assess COVID-19 related KAB, measures developed for use in studies of other global pandemics, for example the 2003 SARS outbreak, the 2009 H1N1 flu pandemic and the 2013-16 Ebola virus pandemic, were consulted and assessed for suitability and adaptation, where possible. Reliable and trusted web sources in the UK (e.g., Public Health England, the National Health Service; NHS) and internationally (e.g., the Centre for Disease Control, the WHO) were also consulted for current, evidence-based knowledge and information relating to the clinical presentation and transmission of COVID-19. Details of the newly devised questions/measures are described below. For consistency of measurement across the study, these newly developed items are incorporated into follow-up waves, as appropriate/necessary, with modest adjustments depending on the most current public health guidance in the UK.

*2.2.5.1. Confidence in response to COVID-19.* Respondents were asked their views as to how well they think each of the following institutions have handled the COVID-19 pandemic: (1) the UK parliament; (2) the UK government; (3) the devolved government in Wales; (4) the devolved government in Scotland; (5) the devolved government in Northern Ireland; (6) your local government (council or local authority); (7) the police; (8) the legal system; (9) political parties; (10) scientists; (11) doctors and other health professionals and (12) pharmaceutical companies. Responses were scored on a 4-point Likert scale ranging from 1 ‘poor’ to 4 ‘very well’, with an ‘I’m not sure’ response also presented.

*2.2.5.2. Views on current and future state of the COVID-19 crisis.*  Data was collected to assess respondents’ views, “*Do you believe that the worst of the COVID-19 crisis in the UK is…*” (1) behind us, (2) happening now, and (3) ahead of us. Respondents were also asked their views on the future likelihood of a pandemic “*How likely do you think it is that there will be another global pandemic within the next 10 years?”* with answers recorded on a visual slider scale ranging from 0 ‘Not at all likely’ – 100 ‘Extremely likely’, centred at 50 ‘Moderately likely’.

*2.2.5.3. COVID-19 Risk behaviours.*

*2.2.5.3.1. Social connection*. Respondents were asked about their engagement in a list of seven daily activities in the past week and asked to indicate, on how many days (response scale 1 ‘Not at all’ to 5 ‘Every day’), they had: (1) met up with friends or extended family who you do not live with outside your home; (2) met up with friends or extended family inside someone’s home or other indoor place; (3) met up with friends or extended family in a private garden? (4) gathered in a group of more than 2 people in a park or public space; (5) when you have met up with others, been inside their home or any other indoor place; (6) stayed at least 2 metres (6ft) away from others when in public; (7) engaged in close contact greetings with other people outside of your home (e.g., shaking hands, kissing, hugging); (8) travelled on public transport (e.g., trains, underground, buses); and (8) worked from home?

*2.2.5.3.2. Hygiene practices.* Respondents were asked to report on whether and how they have changed behaviours relating to their personal care and health recently to reduce their personal risk of being infected by COVID-19 during the pandemic. Statements were posed as follows: “To reduce your risk of being infected by COVID-19 in the past week how often have you…”: (1) Worn a face covering if you are in an enclosed public space (e.g., in a shop, taxi, at a station or on public transport); (2) Washed your hands with soap and water more often than usual; (3) Washed your hands as soon as you returned home after being outside; (4) Washed your hands after touching frequently touched surface or objects, including money and shopping items; (5) Used hand sanitising gel if soap and water were not available; (6) Used disinfectants to wash surfaces in your home more frequently; (7) Avoided touching your eyes, nose or mouth; (8) Sanitized the handles of shopping trolleys or baskets before shopping; (9) Taken steps to avoid sharing items with people you do not live with, such as utensils, dishes, drinks and towels; (10) Avoided close contact greetings with other people outside of your home (e.g., shaking hands, kissing, hugging; (11) Taken steps to avoid close contact with a person who is ill; (12) Stayed at least 2 metres (6ft) away from others when in public; and (13) Covered your nose and mouth with a tissue or sleeve when coughing or sneezing. Response categories were no, occasionally, or whenever possible. Responses ranged from 1 ‘Never’ to 3 ‘Whenever possible’.

*2.2.5.4. Competency, opportunity, and motivation to engage in health behaviours to reduce risk of COVID-19 transmission**.* Seventeen questions based on the COM-B (Capability, Opportunity, Motivation-Behaviour) model of behaviour change (Michie, Van Stralen, & West, 2011) assessed respondents’ ability to engage in *social distancing’*. Questions focused on respondents’ perception of the extent to which they experienced sufficient motivation, capability, and opportunity to enact the recommended behaviours. Items were adapted from a preliminary version of the COM-B self-evaluation questionnaire (COM-B-Qv1) (Michie, Atkins, & West, 2014). Participants were asked to indicate the extent to which eighteen statements were true for them during the COVID-19 pandemic on a 5-point Likert scale ranging from 1 ‘strongly disagree’ to 5 ‘strongly agree’. S*ocial distancing* was defined as ‘e.g., not entering other people’s homes, maintaining a 2 metre distance from people, working from home, etc.) during the pandemic. **Capability** was measured by three items: “I knew about why it was important and had a clear idea about how the virus was transmitted”, “I knew about how and when to do it” and “I was able to overcome the physical and/or mental barriers that might have stopped me from doing it”. **Opportunity** was measured by seven items, split into physical opportunity: “I had the necessary time and facilities to do it”, “It was easy for me to do it”, “People were doing it around me”, “I had reminders that prompted me” and social opportunity: “I had support from others”, “I felt like doing it was normal and expected” and “I felt like people would disapprove if I didn’t do it”. **Motivation** was measured by eight items, split into five items measuring reflective motivation: “I intended to do it”, “I felt that I wanted to do it”, “I believe that it was a good thing to do”, “I developed a specific plan for doing it”, “I developed a habit of it in my everyday routine” and three items measuring automatic motivation: “It made me feel bored, tired, anxious or lonely”, “I would feel bad if I didn’t do it” and “I felt like I could control or cope with how it made me feel so I could do it”.

*2.2.5.5. Experiences of COVID-19 infection - self.* Respondents were asked “*To the best of your knowledge, to date, have you been infected by COVID-19?*” (I’m not sure/yes/no response categories). Three additional statements were presented to those responding ‘I’m not sure’ to clarify their response, as follows: “I have had symptoms but I do not think I have been infected with the COVID-19 virus”, “I have not had symptoms but I still think I have been infected with the COVID-19 virus”, or “I have had symptoms and I think I may have been infected with the COVID-19 virus however I have not been tested” (selection of one option only permitted).

For those indicating a ‘yes’ response, a series of additional questions were asked. First, two statements were presented to clarify their testing history: “I have had symptoms and I think I have been infected with the COVID-19 virus, however I have not been tested” or “Yes, I have been tested for COVID-19 and the test was positive”. Respondents who indicated that they had been infected, or were no sure about their infection status were then asked to report (1) “How unwell did you feel?”, with responses recorded on a visual slider scale ranging from 0 ‘Not at all unwell’ to 100 ‘Extremely unwell’, centred at 50; and (2) whether they were admitted to hospital (Yes/No response).

*2.2.5.6. Experiences of COVID-19 infection – household/extended family*. Respondents were asked “*Has anyone else from your household or (separately) extended family been diagnosed with COVID-19 (confirmed by test)?”* (Yes, No, Not applicable – live alone/Not applicable).

*2.2.5.7. Experiences of self-isolation.* Respondents were presented with a definition of self-isolation (‘*self-isolation means that you have COVID-19 symptoms, or if someone you live with has symptoms, you must not leave your home for between 7-14 days’*) and were asked whether they had self-isolated during the COVID-19 pandemic (Yes/No response).

Those with children under 18 years of age living in their household were also asked two additional questions in relation to experiences of self-isolation: (1) *Have one or more of the children in your household (under the age of 18) been asked to self-isolate because they have tested positive for COVID-19?*; and (2) *Have one or more of the children in your household (under the age of 18) been asked to self-isolate because one of their close contacts has tested positive for COVID-19?*”. Both statements scored using a yes/no/don’t know response scale.

*2.2.5.8. Perceived risk of contracting COVID-19.* Respondents estimated on a visual slider (ranging from 0% ‘No risk’ on the left-hand side to 100% ‘Great risk’ on the right-hand side, centred at 50% ‘Moderate risk’) their personal percentage risk of becoming infected with COVID-19 over the next month. Respondents were also asked to consider “If you were to become infected (or reinfected) with COVID-19 within the next month, how severe do you think your symptoms would be?”, with a visual slider scale representing ‘severity of symptoms’ ranging from 0 ‘Not severe’ to 100 ‘Very severe’, centred at 50 ‘Moderately severe’.

*2.2.5.9. Anxiety and threat relating to COVID-19.* Respondents’ degree of anxiety about the COVID-19 pandemic was assessed using a single visual slider scale, ranging from 0 ‘not at all anxious’ on the left-hand side to 100 ‘extremely anxious’ on the right-hand side (centred at 50, somewhat anxious). Separately, respondents recorded their view on the statement, “*How personally threatened do you feel by COVID-19*?” using a visual slider scale ranging from 0, not at all threatened on the left-hand side to 100, extremely threatened on the right-hand side, centred at 50.

*2.2.5.10. COVID-19 vaccine.* At the time this survey was designed, the vaccination rollout had been in operation for approximately three months (commencing on 8 December 2020). Respondents were asked ‘*Have you been vaccinated for COVID-19?*’ (Yes/No response). For those answering ‘no’, a follow-up question was asked ‘*Multiple vaccines for COVID-19 have now been developed. Will you take a vaccine for COVID-19 when it becomes available to you?’ (*Yes, No, Maybe response options). For those answering, ‘Yes or Maybe’, a follow-up question was posed: ‘*Do you have a preference for which COVID-19 vaccine you would receive?*’ with responses (1) Pfizer/BioNTech vaccine; (2) Oxford/AstraZeneca vaccine; (3) Moderna vaccine; or (4) I don’t have any preference.

*2.2.5.11. Capability, opportunity, and motivation to take a COVID-19 vaccine.* All respondents who said ‘no’ or ‘maybe’ to accepting the vaccine were provided with the following question, ‘When it comes to you personally having the COVID-19 vaccine, what do you think it would take for you to have it?’ and asked to consider the following statements, responding on a 5-point Likert scale ranging from 1 ‘Strongly disagree’ to 5 ‘Strongly agree’: I would need to… (1) *Know more about why it is important and what benefits it will have*; (2) *Know more about how and when I will get the vaccine*; (3) *Be able to overcome the physical barriers that might stop me from doing it* (e.g., ability to attend an appointment); (4) *Be able to overcome mental barriers that might stop me from doing it* (e.g., reduce worry about having the vaccine); (5) *Have more time to do it*; (6) *Have easier access for me to do it* (e.g., to local facilities); (7) *Have more people doing it around me*; (8) *Have more triggers or reminders to prompt me to do it*; (9) *Have more support from others*; (10) *Feel that I want to do it enough* (e.g., feel a sense of satisfaction from doing it); (11) *Feel that I need to do it enough* (e.g., care more about the negative consequences of not doing it); (12) *Believe that it is a good thing to do* (e.g., have a strong sense that you should do it); (13) *Develop better plans for doing it* (e.g., have a clear plan for doing it); (14) *Trust that it was safe for me to do*; and (15) *Have a choice about which brand of vaccine I receive*.

*2.2.5.12. Family and friend COVID-19 vaccination*. Respondents were then asked, ‘*Have any of your family or friends received the COVID-19 vaccine?*’ (Yes/No/Not applicable response). For those indicating yes, the question was posed ‘*Although it is relatively rare, some people can have adverse reactions to vaccines. Did any of your family members or friends have an adverse reaction to the COVID-19 vaccine?*’ (Yes/No/Don’t know responses).

*2.2.5.13. Child COVID-19 vaccination.* All respondents were presented with the statement: ‘M*ultiple vaccines for COVID-19 have now been developed. Will you give your child a vaccine for COVID-19 when it becomes available?’* (Response options: Not applicable – I don’t have children under 18; Yes; Maybe; No; My child has already received the vaccine).

*2.2.5.14. COVID-19 vaccine beliefs.* All respondents were asked to report their beliefs about each of the three COVID-19 vaccines approved for use in the UK by March 2021. The following statements were presented separately for each vaccine: ‘The ‘Pfizer/BioNTech vaccine| Oxford/AstraZeneca vaccine| Moderna vaccine is effective’ and ‘The ‘Pfizer/BioNTech vaccine| Oxford/AstraZeneca vaccine| Moderna vaccine is safe’, with visual sliders used to record beliefs ranging from 0 ‘Completely disagree’ to 100 ‘Completely agree’, centred at 50.

*2.2.5.15. Attitude towards mandatory vaccination*. Respondents were asked whether they agree (yes/no response) to this statement: “*The UK government should make it mandatory that citizens take a vaccine for COVID-19*.”

*2.2.5.16. Vaccine conspiracy beliefs.* Five statements were presented to assess respondents’ beliefs around COVID-19 vaccines, using visual sliders ranging from 0 ‘Not at all’ to 100 ‘Completely’, centred at 50, as follows: (1) *The vaccines contain a microchip*; (2) *The vaccines will alter your DNA*; (3) *The vaccines will give you COVID-19*; (4) *The vaccines are not safe because they were rapidly developed and tested*; and (5) The *vaccines can damage fertility*.

*2.2.5.17. Science conspiracy beliefs.* Four statements were presented to assess respondents’ beliefs about claims made by healthcare professionals and scientists (responses scored on a visual slider ranging from 0 ‘Not at all’ to 100 ‘Completely’, centred at 50): (1) *They often deceive or mislead the public*; (2) *They often cover up their mistakes*; (3) *They are more concerned with making money than taking care of people*; and (4) *They don’t know what they are doing*.

*2.2.5.18. Life after the pandemic.* Respondents were asked a series of questions to determine how they expect to behave once the pandemic is over: “*We would now like to ask you to think about whether you expect your life to return to how it was before, once the pandemic is over:*

*After the pandemic, do you expect*…” Respondents were presented with 12 behaviours and asked whether they expected to engage in them more or less after the pandemic with responses scored on a 5-point Likert scale ranging from (1) Much less to (5) Much more.

(1) To spend money each month (2) To try and save money each month (3) To travel to work (4) To eat out in cafes and restaurants (5) To shop online; (6) To travel abroad; (7) To keep your distance from other people (social distancing) in public; (8) To pay attention to hygiene (e.g., hand washing); (9) To socialise with friends; (10) To attend large social gatherings (e.g., concerts or sports events); (11) To make close contact greetings (e.g., handshakes or hugs); and (12) To see your family members.

**2.2.6. Socio-political module.**

*2.2.6.1 European Referendum.* A series of questions were asked to obtain respondents’ views on the European Referendum held in 2016. The first question, adapted from a 2020 YouGov political poll (Smith, 2020), asked respondents to consider: “*In hindsight, do you think Britain was right or wrong to vote to leave the EU?,* with responses scored on a 7-point Likert scale ranging from (1) very wrong to (7) very right. Next, a series of questions were generated to assess respondents their views on the UK’s future after the process of leaving the EU was completed in 2020. Seven statements were presented as follows: At the end of December 2020, Britain finished its process of leaving the EU. The following questions are about how you think this has affected the UK. Since leaving the EU, do you think*… (1) the UK’s economic position is… (2) The UK’s international political standing is… (3) The UK’s ability to control immigration and the border is… (4) The UK’s trade deals are… (5) The UK’s ability to respond effectively to international outbreaks of disease is… (6) The UK’s ability to respond to security threats is… and (7) The relationship between the countries in the UK (England, Wales, Scotland, and Northern Ireland) is…* with respondents’ answers scored using a 5-point Likert scale ranging from 1, a lot worse to 5 much better.

*2.2.6.2. Family/friends disharmony: political and COVID-19 beliefs.* Respondents were presented with the statement: ‘*Sometimes political differences can cause families and friends to fall out with each other, so that they avoid talking to each other or even stop talking to each other altogether. We would like to know whether this has happened to you. Have you fallen out with other family members or (separately) friends over…* (1) their political views; (2) the issue of Brexit; (3) issues relating to the pandemic (e.g., vaccination, social distancing); (4) their belief in conspiracy theories; and (5) any other reason. Response options were as follows: No; It has become more awkward to speak to some in my family/some of my friends; I have stopped talking to some family members/some of my friends altogether; or Not applicable.

*2.2.6.3 Voting behaviour.* All respondents were also asked to indicate to which party they would give their 1^st^ preference vote to if a general election were to be called tomorrow: (1) I am not eligible to vote; (2) I would not vote, (3) Alliance Party of Northern Ireland; (4) BREXIT Party; (5) Conservative and Unionist Party; (6) Democratic Unionist Party (DUP); (7) Green Party; (8) Labour Party; (9) Liberal Democrats; (10) Plaid Cymru; (11) Scottish National Party (SNP); (12) Sinn Féin; (13) Social Democratic and Labour Party (SDLP); (14) UK Independence Party (UKIP); (15) Ulster Unionist Party; or (16) Other.

*2.2.6.4. Trust.* Respondents were asked the extent to which they have trust in the following institutions/groups: (1) UK parliament; (2) the UK government; (3) the devolved government in Wales; (4) the devolved government in Scotland; (5) the devolved government in Northern Ireland; (6) your local government (council or local authority); (7) the police; (8) the legal system; (9) political parties; (10) scientists; (11) doctors and other health professionals and (12) pharmaceutical companies. Responses were scored on a 5-point Likert scale ranging from 1 ‘do not trust at all’ to 5 ‘completely trust’.

*2.2.6.5. Left- and Right-Wing Authoritarianism.* In order to assess both left- and right-wing attitudes, a combined measure was created using items taken and modified from separate left- and right-wing scales. Right-wing attitudes were from the *Very Short Authoritarianism Scale (VSA)* (Bizumic & Duckitt, 2018). Under the Dual-Process Motivational Model (Duckitt, 2001, 2009), right-wing authoritarianism (RWA) and social dominance orientation (SDO) are conceptualised as value-attitude-belief dimensions which emerge from two different motivational schemas: threat-control (RWA) and competition-dominance (SDO). Both are robust predictors of a range of right-wing political beliefs, including prejudice. Past research also demonstrates that RWA can interact with the perception of threat to produce support for anti-democratic policies (Cohrs, Maes, Moschner, & Kielmann, 2007; Kossowska et al., 2011). The six-item VSA was used to assess respondents’ levels of RWA and includes items such as: ‘*It’s great that many young people today are prepared to defy authority*’; ‘*What our country needs most is discipline, with everyone following our leaders in unity*’; and ‘*Our society does NOT need tougher government and stricter laws’*. Left-wing attitudes were modified from the *Left-Wing Authoritarianism (LWA) Index* (Costello et al., 2020). LWA has been conceptualised as authoritarianism (e.g. aggression, submission, conventionalism) among individuals who oppose traditional established hierarchies of moral and practical authority(Altemeyer & Altemeyer, 1996). Despite RWA receiving considerably more attention in the political psychology literature, the robust conceptualisation and measurement of LWA has recently been considered (Costello et al., 2020). Six-items, either taken directly from, or modified from the LWA Index were used to assess respondents’ levels of LWA in this study. Items included *‘The rich should be stripped of their belongings and status’; ‘Schools should be required by law to teach children about our country's history of racism, classism, sexism, and homophobia’;* and *‘University authorities are wrong to ban hateful speech from campus’.* All items were scored on a 5-point Likert scale ranging from 1 ‘strongly disagree’ to 5 ‘strongly agree’, with several items reverse coded.

**2.2.7. Mental health and psychological module**

Experiences of mental health difficulties are core outcomes for the C19PRC Study. A key objective of the study was to administer a range of brief, standardised questionnaires to screen for the presence of common mental disorder, which would be repeated across all survey waves. Details are included below.

*2.2.7.1. International Prolonged Grief Disorder Scale (IPGDS)* (Killikelly et al., 2020)*.* Prolonged grief was assessed used a modified version of the IPGDS, a 14-item measure which seeks to operationalise the ICD-11 definition of PGD in a self-report questionnaire format. Firstly, participants are asked if, at any time in their life, if someone close to them (e.g., partner, parent, child, friend) has died. If yes, respondents are then presented with a series of items related to the details of this bereavement: (1) when this death occurred, responses ranging from less than 6 months ago to more than 20 years ago, (2) how old the person was when they died, response as age in years, and (3) if the death was reported to have occurred within the last 2 years, they were asked if the death was related to COVID-19 illness (yes/no response). Respondents who reported a bereavement were then asked to consider how well each of the 14 items best describes how they have been feeling over the past week. Items 1 and 2 assess the two core PGD symptoms, items 3-12 assess emotional pain, item 13 functional impairment and item 14 cultural norms. Response options were scored on a 5-point Likert scale ranging from 1 ‘Not at all’ – 5 ‘Always’. The threshold for clinical diagnosis of PGD is currently under investigation (see <https://www.traumameasuresglobal.com/ipgds>)

*2.2.7.2.* *Patient Health Questionnaire-9 (PHQ-9*) (Kroenke, Spitzer, & Williams, 2001). Depression was assessed with the PHQ-9, a nine-item measure which corresponds to the DSM-IV Diagnostic Criterion A symptoms for major depressive disorder (American Psychiatric Association, 2000a). Participants were asked how often, over the last two weeks, they had been bothered by each of the depressive symptoms. Response options were “not at all”, “several days”, “more than half the days”, and “nearly every day”, scored as 0, 1, 2 and 3, respectively. PHQ-9 scores range from 0 to 27, with scores of ≥5, ≥10, ≥15, representing mild, moderate and severe levels of depression severity (Kroenke et al., 2001). A threshold of ≥10 was used in this study. Psychometric properties of the PHQ-9 are well documented (see Kroenke, Spitzer, Williams, and Löwe (2010) for an overview).

*2.2.7.3. Suicidality.* Following the PHQ-9, the last item of which asks respondents about whether they experienced thoughts of death or self-harm in the last two weeks, respondents were asked if they would be content to answer a series of questions relating to defeat and entrapment, perceived burdensomeness and thwarted belonginess, and thoughts and actions of self-harm and suicide. Respondents who answered ‘No’ were automatically skipped to the next measures (GAD-7, see next sub-section); respondents who answered ‘Yes’ were screened into this section.

*2.2.7.3.1. The Short Defeat and Entrapment Scale (SDES)* (Griffiths et al., 2015). Defeat (conceptualised as a failed social struggle) and entrapment (conceptualised as a perceived inability to escape from aversive situations) (Gilbert & Allan, 1998) are considered to be two distinct, yet highly correlated constructs, considered to be strongly related to a range of negative mental health outcomes in both clinical and non-clinical populations (e.g. depression, suicidal ideation, psychosis) (Taylor, Gooding, Wood, & Tarrier, 2011). Scales typically measure defeat and entrapment separately, however, factor analytic studies typically find that they load onto a single factor (see Griffiths et al. (2015) for review). The SDES was developed to assess both constructs in a single scale, with relatively few items to improve clinical utility (Griffiths et al., 2015). It comprises 4 items to measure defeat (e.g. “I feel defeated by life”) and 4 items to measure entrapment (e.g. “I would like to get away from who I am and start again). Items are scores on a 5-point Likert scale ranging from 0 ‘Not at all’ to 4 ‘Extremely’. The scale has demonstrated high internal consistency and construct validity, strongly correlating with measures of depression, hopelessness, perceived burdensomeness, and thwarted belongingness (Griffiths et al., 2015; Höller et al., 2020).

*2.2.7.3.2. Interpersonal Needs Questionnaire (INQ-15)* (Van Orden, Cukrowicz, Witte, & Joiner Jr, 2012). The INQ-15 was derived from the interpersonal theory of suicide (Joiner, 2007; Van Orden et al., 2010) and was developed to measure thwarted belongingness and perceived burdensomeness. Similar to defeat and entrapment, they are considered to be distinct but related constructs, relevant to both clinical and nonclinical samples (Van Orden et al., 2012). The INQ-15 contains items: 9 which measure perceived belongingness (e.g., “*These days other people care about me*,” reverse coded), and 6 which measure perceived burdensomeness (e.g., “*These days I feel like a burden on the people in my life*”). Respondents are asked to indicate the degree to which each item is true for them when considering their recent thoughts and feelings. Responses are scored on a 7-point Likert scale ranging from 1 ‘Not at all true for me’ to 7 ‘Very true for me’ (items 7, 8, 10, 13, 14, and 15 reverse coded), with higher scores indicating higher levels of thwarted belonginess and perceived burdensomeness (Van Orden et al., 2012). The measure has been validated for use in young adults and has good internal reliability and construct validity (Hallensleben, Spangenberg, Kapusta, Forkmann, & Glaesmer, 2016; Hill et al., 2015; Van Orden et al., 2012).

*2.2.7.3.3. Self-injurious thoughts and behaviours*. Adapted from the 2014 English Adult Psychiatric Morbidity Survey (McManus, Bebbington, Jenkins, & Brugha, 2016)).

Respondents who screened into this section were presented with the following statement to assess lifetime suicidal ideation: “*There may be times in everyone's life when they become very miserable and depressed and may feel like taking drastic action because of these feelings. Have you ever thought of harming yourself or taking your life, even if you would not really do it*?” (Yes/No response). Next, the respondent was asked to re-consider the statement but with specific reference to the time period since the pandemic began (Yes/No response). Respondents were then asked, “*Have you ever made an attempt to take your own life*?” (Yes/No response). Filtering was imposed depending on the respondent’s answer: (1) adults who responded ‘No’ were asked if they had ever deliberately harmed themselves in any way but not with the intention of taking your own life (Yes/No response) and, if yes, was this in (a) the last two weeks and/or (b) in the last year but not in the last two weeks (Yes/No response to both questions); (2) adults who responded ‘Yes’ were asked if the attempt to take their own life happened in (a) the last two weeks and/or (b) in the last year but not in the last two weeks (Yes/No response to both questions), before being asked if they had ever deliberately harmed themselves. At the end of the survey, all respondents were presented with contact information for self-harm or suicide support services.

*2.2.7.4. Generalized Anxiety Disorder Scale (GAD-7)* (Spitzer, Kroenke, Williams, & Löwe, 2006). Experiences of generalized anxiety were assessed using the GAD-7. Respondents were asked to report, on a 4-point Likert scale ranging from 0 (not at all) to 3 (nearly every day), how often in the past 7 days they were bothered by seven anxiety symptoms (e.g. trouble relaxing, becoming easily annoyed or irritable). The GAD-7 was originally validated in a primary care sample and a cut-off score of 10 had a sensitivity value of 0.89 and a specificity value of 0.82 for identifying generalised anxiety disorder (Spitzer et al., 2006), and a threshold of 10 was used in this study. The GAD-7 has demonstrated good reliability and construct validity, as evidenced by strong associations with other established measures of anxiety as well as diagnoses of GAD and its associations with depression, self-esteem, life satisfaction, and resilience (Löwe et al., 2008).

*2.2.7.5. International Trauma Questionnaire (ITQ)* (Cloitre et al., 2018). Post-traumatic stress disorder was assessed using the ITQ, a self-report measure of ICD-11 PTSD based on a total of six symptoms across the three symptom clusters of Re-experiencing, Avoidance, and Sense of Threat; each symptom cluster is comprised of 2 symptoms. Participants were asked to complete the ITQ as follows: “…in relation to your experience of the COVID-19 pandemic, please read each item carefully, then select one of the answers to indicate how much you have been bothered by that problem in the past month”. The PTSD symptoms are accompanied by three items measuring functional impairment caused by these symptoms. All items are answered on a 5-point Likert scale, ranging from 0 (Not at all) to 4 (Extremely) with possible PTSD scores ranging from 0 to 24. A score of ≥ 2 (Moderately) is considered ‘endorsement’ of that symptom. A PTSD diagnosis requires traumatic exposure, and at least one symptom to be endorsed from each PTSD symptom cluster (Re-experiencing, Avoidance, and Sense of Threat), and endorsement of at least one indicator of functional impairment. The psychometric properties of the ITQ scores have been demonstrated in multiple general population (Ben‐Ezra et al., 2018; Cloitre et al., 2019) and clinical and high-risk samples (Hyland et al., 2017; Karatzias et al., 2016; Vallières et al., 2018) samples.

*2.2.7.6. Mood Disorders Questionnaire (MDQ)* (Hirschfeld et al., 2000). The MDQ is a brief, self-report screening instrument for bipolar spectrum disorders with items derived from DSM-IV criteria and clinical experience (Hirschfeld et al., 2000). Respondents are asked “Has there ever been a period of time when you were not your usual self and…” and are presented with 13 yes/no items such as “…you felt much more self-confident than usual?” and “…you felt so good or so hyper that other people thought you were not your normal self or you were so hyper that you got into trouble?”. This is followed by 4 items specifically enquiring about (1) the co-occurrence of these experiences, (2) functional impairment as a result of these experiences, (3) a diagnosis of bipolar or manic-depressive illness in blood relatives and (4) whether the respondent had ever been diagnosed with bipolar or manic-depressive disorder by a healthcare professional. A positive screen for bipolar disorder is given if 7 or more of the 13 yes/no experiences are endorsed, a positive endorsement for these experiences co-occurring and reporting at least moderate impairment. The MDQ was originally validated in a psychiatric outpatient sample and the screening score of 7 or more yielded good sensitivity (0.73) and very good specificity (0.90) (Hirschfeld et al., 2000), although the sensitivity and specificity characteristics of the questionnaire have been found to differ substantially in general population samples (Chung, Tso, & Chung, 2009; Hirschfeld et al., 2003). In the current study, lifetime endorsement of all of the13 mania symptoms was additionally followed up with an item enquiring if that experience occurred within the last year.

*2.2.7.7. Psychosis Screening Questionnaire (PSQ)* (Bebbington & Nayani, 1995). The PSQ is used to assess self-reported psychotic symptoms within the past year. In the current study, however, the PSQ was modified to first assess for lifetime endorsement of these symptoms before enquiring about the presence of these symptoms in the past year (yes/no response) if lifetime experience was endorsed. The PSQ consists of five main questions inquiring about mania, thought insertion, paranoia, strange experiences, and hallucinations (P1–P5) and one or two subsidiary questions (a and b), to corroborate the clinical relevance of this experience. Responses to each experience are scored as (1) Yes, (2) No and (3) Unsure. For example, the main item to screen for paranoia (PSQ3: Have there been times when you felt that people were against you?) must be endorsed before participants are asked the first subsidiary item (PSQ3a: Have there been times when you felt that people were deliberately acting to harm you or your interests?), which in turn must be endorsed before the second subsidiary item is asked (PSQ3b: Have there been times when you felt that a group of people were plotting to cause you serious harm or injury?). The instrument was originally validated in a mixed sample of psychiatric inpatients, psychiatric outpatients and individuals attending their GP surgery. Sensitivity (96.9%) and specificity (95.3%) were found to be high (Bebbington & Nayani, 1995).

*2.2.7.8.* *Short Warwick-Edinburgh Mental Well-being Scale (SWEMWBS)* (Stewart-Brown et al., 2009). The SWEMWBS is a 7-item unidimensional scale which has robust measurement properties for monitoring mental well-being in population surveys. Respondents answered the following questions with reference to the last two-weeks: (1) I’ve been feeling optimistic about the future; (2) I’ve been feeling useful; (3) I’ve been feeling relaxed; (4) I’ve been dealing with problems well; (5) I’ve been thinking clearly; (6) I’ve been feeling close to other people; and (7) I’ve been able to make up my own mind about things. All items are scored on 5-point Likert scale, ranging from 1, none of the time to 5, all of the time. Raw scores range from 7-35, with higher scores indicating overall better mental wellbeing. The total raw scores can then converted into a metric score using the following conversion table: <https://warwick.ac.uk/fac/sci/med/research/platform/wemwbs/using/howto/swemwbs_raw_score_to_metric_score_conversion_table.pdf>. Raw scores need to be converted into metric scores in order for comparisons to be made across different studies.

*2.2.7.9. Hopefulness (Brief-H-Pos Scale)* (Fraser et al., 2014). Respondents were asked to complete the Brief-H-Pos Scale, a two-item measure which is a positive re-framing of the negatively worded two-item Hopeless Scale (Everson et al., 1996). The two items, “The future seems to me to be hopeful and I believe that things are changing for the better” and “I feel that it is possible to reach the goals I would like to strive for”, are scored on a 5-point Likert scale ranging from 1, absolutely disagree to 5, absolutely agree (higher scores indicate higher levels of hopefulness). Fraser et al. (2014) demonstrated that the scale had good internal consistency, test-re-test reliability and concurrent validity (compared to the Beck Hopelessness Scale (Beck, Weissman, Lester, & Trexler, 1974) and the Centre for Epidemiological Studies on Depression Scale (Radloff, 1977)), and recommended the scale as a useful screener for hopefulness in large general population studies.

*2.2.7.10. Loneliness Scale* (Hughes, Waite, Hawkley, & Cacioppo, 2004). Social connectedness was measured using the three-item Loneliness Scale, which was specifically designed for use in large-scaled population surveys (Hughes et al., 2004). Respondents were asked how often they felt: (1) that they lacked companionship; (2) left out; and (3) isolated from others. Responses were scored on a 3-point scale (hardly ever, sometimes, or often).

*2.2.7.11. Happiness* (Office for National Statistics, 2016). A single-item measuring subjective happiness, as included in the Annual Population Survey, was included, as follows: *“Overall, how happy did you feel yesterday, where 0 is ‘not at all happy’ and 10 is ‘completely’ happy?”* Scores of 0-4 are considered to reflect ‘low’ happiness, scores of 5-6 ‘medium’ happiness, 7-8 ‘high’ happiness’ and 9-10 ‘very high’ happiness, levels (Office for National Statistics, 2016).

*2.2.7.12. Daily functioning and wellbeing.* Respondents were asked, ‘*What has been helpful to your wellbeing and day-to-day functioning at home over the last two weeks?* (choose all that apply)’: (1) work; (2) watching television/series; (3) listening to music; (4) gaming; (5) social media; (6) reading a book; (7) sports; (8) chilling; (9) online contact with relatives or friends; (10) being together with the family; (11) playing cards/board games; (12) DIY/crafts; (13) cooking/dining; (14) meditation/mindfulness; (15) something else (specify); or (16) none of the above. Respondents were also asked, ‘*What has been harmful to your wellbeing and day-to-day functioning at home over the last two weeks?* (choose all that apply)’: (1) boredom; (2) conflicts; (3) work; (4) irritations with family members; (5) noise disturbance; (6) loneliness; (7) missing social contact with friends and/or family; (8) worrying about own health; (9) worrying about the health of others; (10) concerns about the coronavirus in general; (11) coronavirus-related news items; (12) something else (specify); or (13) none of the above. These items were adapted from (Janssen et al., 2020).

*2.2.7.13* *Social engagement/contact*. Five questions were administered to assess respondents’ social connectedness, as follows: (1) Do you use social media?; (2) Have you made or received a personal telephone call or similar (Skype, Zoom) in the last week? (3) Have you written or received a personal letter or email in the last week? (4) In the past month, have you attended a meeting or gathering of any club, organisation, society or group in person or virtually?; and (5) Do you have at least one friend living in or near your area?. Response options for all questions (Yes/No).

**2.2.8. Parenting.**

The following module was administered to respondents indicating that they have children under the age of 18 years, regardless as to whether they live primarily with the respondent in their household or elsewhere.

*2.2.8.1. Parenting Scale Short Form (PS-8)* (Kliem et al., 2019). Parenting style was measured using the PS-8. The scale consists of 8 items that measure self-assessed parenting response to problematic child behaviour during the past two months. Responses are scored on a 7-point Likert scale, anchored between two alternative responses to a particular situation (e.g. “When I give a fair threat or warning…” scored (1) I often don’t carry it out – (7) I always do what I said). These 8 items reflect two subscales: laxness and over reactivity. The original PS scale consisted of 30 items with an additional ‘verbosity’ subscale, however, this factor could not be replicated in several studies, prompting the creation of the short form scale. The psychometric properties of the PS-8 were found to be satisfactory: good internal consistency was reported for both mothers and fathers on both subscales and moreover, PS-8 mean score was associated with self-reported parental anxiety and depression (Kliem et al., 2019).

*2.2.8.2.* *Home-schooling*. Parents of children under 18 and respondents who reported having children under 18 living in their home, were asked to consider how much time they spent on an average day during the past two weeks home-schooling one or more children Responses were recorded in hours.

*2.2.8.3. Parental warmth and criticism.* Two items, adapted from Janssen et al. (2020), asked parents of under 18s to consider (i) *how warm/loving they were towards their child/children* and (ii) *how critical they were towards their child/children* throughout the past two weeks. Responses to both items were scored on a 5-point Likert scale ranging from 1 ‘Never’ to 5 ‘Always’.

**2.2.9. Health.**

*2.2.9.1 General health* (Contoyannis, Jones, & Rice, 2004). A single-item taken from the British Household Panel Survey asked respondents to considered “*Compared to someone your own age, would you say your health has on the whole been?,* with responses recorded on a 5-point Likert scale ranging from 1, poor to 5, excellent.

*2.2.9.2. Treatment seeking behaviour.* All respondents were asked about their history of mental health difficulties. The following statement was presented: “Mental health difficulties are very common. It will help us understand our survey results if you would tell us whether you currently or have in the past received treatment (medication or talking therapies) for these kind of difficulties”, along with the following response categories: (1) I never received treatment for mental health problems; (2) I have received treatment for mental health problems in the past; (3) I am currently receiving treatment for mental health problems; (4) I am currently receiving treatment for mental health problems, but it has been cancelled temporarily due to the lockdown; (5) I am currently on a waiting list to receive treatment for a mental health problem; and (5) I’d prefer not to answer this question. For those currently receiving mental health treatment, an additional question was asked ‘How are you currently receiving this treatment?’, with the following response options: (1) by face-to face appointments; (2) by online consultations; or (3) both modes.

*2.2.9.3. Health service use.* Respondents were asked to indicate their use of a range of healthcare services since the beginning of the pandemic. A list of services were provided as follows: (1) dentist; (2) sight test; (3) hearing test; (4) other health check-ups; (5) physiotherapist; (6) occupational therapist; (7) chiropodist; (8) social worker; (9) speech therapist; (10) day centre; (11) outpatient services; (12) inpatient services; (13) GP appointment. For each service, four response options were provided: (1) Yes, I had a consultation in person; (2) Yes, I had a consultation online or by telephone; (3) No, the pandemic prevented me from doing so; and (4) No, I didn’t need this service during the pandemic.

**2.2.10. Health-related behaviours.**

*2.2.10.1. Weight.* Respondents were asked to classify their weight according to one of four categories: obese, overweight, normal weight, or underweight. Respondents were also asked to report on whether their weight had changed over the past year on a 7-point Likert scale from (1) I am a lot lighter to (7) I am a lot heavier.

*2.2.10.2. Alcohol Use.* Respondents were asked to complete an adapted version of the 3-item AUDIT-C, which has good specificity and sensitivity for detecting alcohol dependence and at-risk drinking in the general population (Dawson, Grant, Stinson, & Zhou, 2005). The AUDIT-C questions were asked ‘During the last six months: (1) how often did you have a drink containing alcohol (response categories 0 ‘never’ to 4 ‘4 or more times a week’); (2) how many drinks containing alcohol did you have on a typical day when you were drinking (response categories 1 ‘1-2 drinks’ to 5 ’10 or more’; and (3) how often did you have six or more alcohol drinks on one occasion (response categories 1 ‘never’ to 5 ‘almost daily’). Items (2) and (3) were only asked if the respondent did not indicate ‘never’ to item (1).

*2.2.10.3. Sleep Disorders Symptom Checklist -17 (SDS-CL-17)* (Klingman, Jungquist, & Perlis, 2017). The SDS-CL-17 was developed as a screening instrument for six separate categories of sleep disorders: (i) insomnia (ii) circadian rhythm sleep-wake disorders (iii) narcolepsy, (iv) obstructive sleep apnoea (OSA) (v) restless leg syndrome/periodic limb movement disorder (RLS/PLMD) and (vi) parasomnias. Items and responses are based on DSM-IV diagnostic criteria (American Psychiatric Association, 2000b), the International Classification of Sleep Disorders (ICSD)-2 (American Academy of Sleep Medicine, 2005) and expert opinion. Each of the 17 items are framed as a statement with reference to experiencing this in the last year (e.g. “*I am awake 30 minutes or more during the night”*). The frequency each experience is scored on a 5-point Likert scale ranging from ‘never’ to ‘frequently (>3x/week)’. Subscale scores and positive screen cut-off scores can be determined by summing the appropriate responses for each subscale (see Klingman et al. (2017)). Sensitivity and specificity for the disorders ranged from 0.64 – 0.88 (Klingman et al., 2017).

**2.2.11. Adverse Childhood Experiences (ACEs)**

*2.2.11.1. ACE Scale (Felitti et al., 1998)*. A final section was included at the end of the survey relating to adverse or traumatic childhood experiences. Participants were informed of this and asked whether they wished to proceed with this section of the survey; if they did not consent, they were skipped to the debriefing sheet at the end of the survey. Individuals who consented to this section were presented with seven adverse experiences and were asked to consider if they had experienced any of these growing up, prior to their 18^th^ birthday. Respondents were also informed that they could skip any of these items if they did not want to answer. These seven items were adapted from Felitti et al. (1998) and related to experiences of childhood maltreatment and family dysfunction: (1) parental emotional abuse, (2) emotional neglect, (3) physical neglect, (4) parental separation or divorce, (5) living with someone with an alcohol use problem, (6) living with someone with mental illness, and (7) a household member went to prison (yes/no responses). A free-text box also allowed respondents to report any other ACEs if they wished. Three of the *ACE Scale* items related to childhood physical abuse, sexual abuse and maternal domestic abuse could not be included in the survey as they were deemed to be incriminating by the survey provider *Qualtrics.* Further, small modifications had to be made to the physical neglect and living with a problem drinker items, to remove incriminating references to drug use.

**References**

Adler, N. E., Epel, E. S., Castellazzo, G., & Ickovics, J. R. (2000). Relationship of subjective and objective social status with psychological and physiological functioning: Preliminary data in healthy, White women. *Health Psychology, 19*(6), 586.

Altemeyer, R. A., & Altemeyer, B. (1996). *The authoritarian specter*. Cambridge: Harvard University Press.

American Academy of Sleep Medicine. (2005). *International Classification of Sleep Disorders: Diagnostic and Coding Manual 2.* Westchester: American Academy of Sleep Medicine.

American Psychiatric Association. (2000a). *Diagnostic criteria from dsM-iV-tr*: American Psychiatric Pub.

American Psychiatric Association. (2000b). *Quick reference to the diagnostic criteria from DSM-IV-TR*: APA Washington, DC.

Bebbington, P., & Nayani, T. (1995). The psychosis screening questionnaire. *International Journal of Methods in Psychiatric Research*.

Beck, A. T., Weissman, A., Lester, D., & Trexler, L. (1974). The measurement of pessimism: the hopelessness scale. *Journal of consulting and clinical psychology, 42*(6), 861.

Bell, D. N., & Blanchflower, D. G. (2021). Underemployment in the United States and Europe. *ILR Review, 74*(1), 56-94.

Ben‐Ezra, M., Karatzias, T., Hyland, P., Brewin, C. R., Cloitre, M., Bisson, J. I., . . . Shevlin, M. (2018). Posttraumatic stress disorder (PTSD) and complex PTSD (CPTSD) as per ICD‐11 proposals: A population study in Israel. *Depression and Anxiety, 35*(3), 264-274. doi:doi:10.1002/da.22723

Bizumic, B., & Duckitt, J. (2018). Investigating Right Wing Authoritarianism with a Very Short Authoritarianism scale. *Journal of Social and Political Psychology*.

Blanchflower, D. G., & Clark, A. E. (2020). *Children, unhappiness and family finances. GLO Discussion paper, No. 561.* Retrieved from Essen, Germany: <https://cpb-us-e1.wpmucdn.com/sites.dartmouth.edu/dist/5/2216/files/2020/09/GLO-DP-05611.pdf>

Cabinet Office. (2015). *Community Life Survey Technical Report 2014-15.* Retrieved from <https://assets.publishing.service.gov.uk/government/uploads/system/uploads/attachment_data/file/470407/Community_Life_2014-15_Combined_technical_report_FINAL.pdf>

Chung, K.-F., Tso, K.-C., & Chung, R. T.-Y. (2009). Validation of the Mood Disorder Questionnaire in the general population in Hong Kong. *Comprehensive psychiatry, 50*(5), 471-476. doi:10.1016/j.comppsych.2008.10.001

Cloitre, M., Hyland, P., Bisson, J. I., Brewin, C. R., Roberts, N., Karatzias, T., & Shevlin, M. (2019). ICD-11 PTSD and complex PTSD in the United States: a population-based study. *Journal of Traumatic Stress*.

Cloitre, M., Shevlin, M., Brewin, C. R., Bisson, J. I., Roberts, N. P., Maercker, A., . . . Hyland, P. (2018). The International Trauma Questionnaire: development of a self‐report measure of ICD‐11 PTSD and complex PTSD. *Acta Psychiatrica Scandinavica, 138*(6), 536-546. doi:10.1111/acps.12956

Cohrs, J. C., Maes, J., Moschner, B., & Kielmann, S. (2007). Determinants of human rights attitudes and behavior: A comparison and integration of psychological perspectives. *Political Psychology, 28*(4), 441-469.

Contoyannis, P., Jones, A. M., & Rice, N. (2004). The dynamics of health in the British Household Panel Survey. *Journal of Applied Econometrics, 19*(4), 473-503.

Costello, T. H., Bowes, S. M., Stevens, S. T., Waldman, I. D., Tasimi, A., & Lilienfeld, S. O. (2020). Clarifying the Structure and Nature of Left-Wing Authoritarianism. *Journal of Personality and Social Psychology*. doi:10.1037/pspp0000341

Dawson, D. A., Grant, B. F., Stinson, F. S., & Zhou, Y. (2005). Effectiveness of the derived Alcohol Use Disorders Identification Test (AUDIT‐C) in screening for alcohol use disorders and risk drinking in the US general population. *Alcoholism: Clinical and Experimental Research, 29*(5), 844-854. doi:10.1097/01.ALC.0000164374.32229.A2

Duckitt, J. (2001). A dual-process cognitive-motivational theory of ideology and prejudice. In *Advances in experimental social psychology* (Vol. 33, pp. 41-113): Elsevier.

Duckitt, J. (2009). Authoritarianism and dogmatism. *Handbook of individual differences in social behavior, 298317*.

Everson, S. A., Goldberg, D. E., Kaplan, G. A., Cohen, R. D., Pukkala, E., Tuomilehto, J., & Salonen, J. T. (1996). Hopelessness and risk of mortality and incidence of myocardial infarction and cancer. *Psychosomatic Medicine, 58*(2), 113-121.

Felitti, V. J., Anda, R. F., Nordenberg, D., Williamson, D. F., Spitz, A. M., Edwards, V., & Marks, J. S. (1998). Relationship of childhood abuse and household dysfunction to many of the leading causes of death in adults: The Adverse Childhood Experiences (ACE) Study. *American journal of preventive medicine, 14*(4), 245-258.

Food and Agricultural Organisation of the United Nation. (2016). *Methods for estimating comparable rates of food insecurity experienced by adults throughout the world.* Retrieved from Rome, Italy.:

Fraser, L., Burnell, M., Salter, L. C., Fourkala, E.-O., Kalsi, J., Ryan, A., . . . Menon, U. (2014). Identifying hopelessness in population research: a validation study of two brief measures of hopelessness. *BMJ Open, 4*(5). doi:10.1136/bmjopen-2014-005093

Gilbert, P., & Allan, S. (1998). The role of defeat and entrapment (arrested flight) in depression: an exploration of an evolutionary view. *Psychological Medicine, 28*(3), 585-598. doi:10.1017/S0033291798006710

Griffiths, A. W., Wood, A. M., Maltby, J., Taylor, P. J., Panagioti, M., & Tai, S. (2015). The development of the short defeat and entrapment scale (SDES). *Psychological Assessment, 27*(4), 1182-1194. doi:10.1037/pas0000110

Hallensleben, N., Spangenberg, L., Kapusta, N., Forkmann, T., & Glaesmer, H. (2016). The German version of the Interpersonal Needs Questionnaire (INQ)–Dimensionality, psychometric properties and population-based norms. *Journal of Affective Disorders, 195*, 191-198.

Hill, R. M., Rey, Y., Marin, C. E., Sharp, C., Green, K. L., & Pettit, J. W. (2015). Evaluating the Interpersonal Needs Questionnaire: Comparison of the reliability, factor structure, and predictive validity across five versions. *Suicide and Life‐Threatening Behavior, 45*(3), 302-314.

Hirschfeld, R. M., Holzer, C., Calabrese, J. R., Weissman, M., Reed, M., Davies, M., . . . Lewis, L. (2003). Validity of the mood disorder questionnaire: a general population study. *American Journal of Psychiatry, 160*(1), 178-180. doi:10.1176/appi.ajp.160.1.178

Hirschfeld, R. M., Williams, J. B., Spitzer, R. L., Calabrese, J. R., Flynn, L., Keck Jr, P. E., . . . Rapport, D. J. (2000). Development and validation of a screening instrument for bipolar spectrum disorder: the Mood Disorder Questionnaire. *American Journal of Psychiatry, 157*(11), 1873-1875. doi:10.1176/appi.ajp.157.11.1873

Höller, I., Teismann, T., Cwik, J. C., Glaesmer, H., Spangenberg, L., Hallensleben, N., . . . Juckel, G. (2020). Short defeat and entrapment scale: A psychometric investigation in three German samples. *Comprehensive psychiatry, 98*, 152160. doi:10.1016/j.comppsych.2020.152160

Hughes, M. E., Waite, L. J., Hawkley, L. C., & Cacioppo, J. T. (2004). A short scale for measuring loneliness in large surveys: Results from two population-based studies. *Research on Aging, 26*(6), 655-672. doi:10.1177/0164027504268574

Hyland, P., Shevlin, M., Brewin, C. R., Cloitre, M., Downes, A., Jumbe, S., . . . Roberts, N. (2017). Validation of post‐traumatic stress disorder (PTSD) and complex PTSD using the International Trauma Questionnaire. *Acta Psychiatrica Scandinavica, 136*(3), 313-322. doi:10.1111/acps.12771

Janssen, L. H., Kullberg, M.-L. J., Verkuil, B., van Zwieten, N., Wever, M. C., van Houtum, L. A., . . . Elzinga, B. M. (2020). Does the COVID-19 pandemic impact parents’ and adolescents’ well-being? An EMA-study on daily affect and parenting. *PLoS one, 15*(10), e0240962. doi:10.1371/journal.pone.0240962

Joiner, T. (2007). *Why people die by suicide*: Harvard University Press.

Karatzias, T., Shevlin, M., Fyvie, C., Hyland, P., Efthymiadou, E., Wilson, D., . . . Cloitre, M. (2016). An initial psychometric assessment of an ICD-11 based measure of PTSD and complex PTSD (ICD-TQ): Evidence of construct validity. *Journal of Anxiety Disorders, 44*, 73-79. doi:10.1016/j.janxdis.2016.10.009

Killikelly, C., Zhou, N., Merzhvynska, M., Stelzer, E.-M., Dotschung, T., Rohner, S., . . . Maercker, A. (2020). Development of the International Prolonged Grief Disorder Scale for the ICD-11: measurement of core symptoms and culture items adapted for Chinese and German-speaking samples. *Journal of Affective Disorders, 277*, 568-576. doi:10.1016/j.jad.2020.08.057

Kliem, S., Lohmann, A., Mößle, T., Foran, H. M., Hahlweg, K., Zenger, M., & Brähler, E. (2019). Development and validation of a parenting scale short form (PS-8) in a representative population sample. *Journal of Child and Family Studies, 28*(1), 30-41. doi:10.1007/s10826-018-1257-3

Klingman, K. J., Jungquist, C. R., & Perlis, M. L. (2017). Introducing the sleep disorders symptom checklist-25: a primary care friendly and comprehensive screener for sleep disorders. *Sleep Medicine Research, 8*(1), 17-25. Retrieved from <https://kmbase.medric.or.kr/KMID/1034320170080010017>

Kossowska, M., Trejtowicz, M., de Lemus, S., Bukowski, M., Van Hiel, A., & Goodwin, R. (2011). Relationships between right‐wing authoritarianism, terrorism threat, and attitudes towards restrictions of civil rights: A comparison among four European countries. *British Journal of Psychology, 102*(2), 245-259.

Kroenke, K., Spitzer, R. L., & Williams, J. B. (2001). The PHQ‐9: validity of a brief depression severity measure. *Journal of General Internal Medicine, 16*(9), 606-613. doi:10.1046/j.1525-1497.2001.016009606.x

Kroenke, K., Spitzer, R. L., Williams, J. B., & Löwe, B. (2010). The patient health questionnaire somatic, anxiety, and depressive symptom scales: A systematic review. *General hospital psychiatry, 32*(4), 345-359.

Löwe, B., Decker, O., Müller, S., Brähler, E., Schellberg, D., Herzog, W., & Herzberg, P. Y. (2008). Validation and standardization of the Generalized Anxiety Disorder Screener (GAD-7) in the general population. *Medical care*, 266-274.

McManus, S., Bebbington, P., Jenkins, R., & Brugha, T. (2016). *Mental health and wellbeing in England: Adult psychiatric morbidity survey 2014.* Retrieved from Leeds:

Michie, S., Atkins, L., & West, R. (2014). The behaviour change wheel: a guide to designing interventions. *Needed: physician leaders, 26*, 146.

Michie, S., Van Stralen, M. M., & West, R. (2011). The behaviour change wheel: a new method for characterising and designing behaviour change interventions. *Implementation science, 6*(1), 42.

Office for National Statistics. (2016). Personal well-being in the UK: 2015 to 2016. Personal well-being findings from the Annual Population Survey (APS), with analysis by country, region and individual characteristics. . Retrieved from <https://www.ons.gov.uk/peoplepopulationandcommunity/wellbeing/bulletins/measuringnationalwellbeing/2015to2016>

Radloff, L. S. (1977). The CES-D scale: A self-report depression scale for research in the general population. *Applied psychological measurement, 1*(3), 385-401.

Smith, F. (2020). With the transition period almost over, by 51% to 40% Britons think we were wrong to vote to leave. Retrieved from <https://yougov.co.uk/topics/politics/articles-reports/2020/12/22/transition-period-almost-over-51-40-britons-think->

Spitzer, R. L., Kroenke, K., Williams, J. B., & Löwe, B. (2006). A brief measure for assessing generalized anxiety disorder: the GAD-7. *Archives of Internal Medicine, 166*(10), 1092-1097. doi:10.1001/archinte.166.10.1092

Stewart-Brown, S., Tennant, A., Tennant, R., Platt, S., Parkinson, J., & Weich, S. (2009). Internal construct validity of the Warwick-Edinburgh mental well-being scale (WEMWBS): a Rasch analysis using data from the Scottish health education population survey. *Health and Quality of Life Outcomes, 7*(1), 1-8. doi:10.1186/1477-7525-7-15

Taylor, P. J., Gooding, P., Wood, A. M., & Tarrier, N. (2011). The role of defeat and entrapment in depression, anxiety, and suicide. *Psychological bulletin, 137*(3), 391-420. doi:10.1037/a0022935

UK Cabinet Office/Department of Education. (2020). Guidance: Critical workers who can access schools or educational settings. Retrieved from <https://www.gov.uk/government/publications/coronavirus-covid-19-maintaining-educational-provision/guidance-for-schools-colleges-and-local-authorities-on-maintaining-educational-provision>

Vallières, F., Ceannt, R., Daccache, F., Abou Daher, R., Sleiman, J., Gilmore, B., . . . Hyland, P. (2018). ICD‐11 PTSD and complex PTSD amongst Syrian refugees in Lebanon: the factor structure and the clinical utility of the International Trauma Questionnaire. *Acta Psychiatrica Scandinavica, 138*(6), 547-557. doi:10.1111/acps.12973

Van Orden, K. A., Cukrowicz, K. C., Witte, T. K., & Joiner Jr, T. E. (2012). Thwarted belongingness and perceived burdensomeness: construct validity and psychometric properties of the Interpersonal Needs Questionnaire. *Psychological Assessment, 24*(1), 197-215. doi:10.1037/a0025358

Van Orden, K. A., Witte, T. K., Cukrowicz, K. C., Braithwaite, S. R., Selby, E. A., & Joiner Jr, T. E. (2010). The interpersonal theory of suicide. *Psychological review, 117*(2), 575.
